# On the Efficacy of Water Transport in Leaves. A Coupled Xylem-Phloem Model of Water and Solute Transport

**DOI:** 10.3389/fpls.2021.615457

**Published:** 2021-02-04

**Authors:** Gen Sakurai, Stanley J. Miklavcic

**Affiliations:** ^1^Institute for Agro-Environmental Sciences, National Agriculture and Food Research Organization, Tsukuba, Japan; ^2^Phenomics and Bioinformatics Research Centre, University of South Australia, Mawson Lakes, SA, Australia

**Keywords:** leaf architecture, vein angle, leaf conductivity, turgor pressure, water transport, sucrose transport, phloem-xylem interactions

## Abstract

In this paper, we present and use a coupled xylem/phloem mathematical model of passive water and solute transport through a reticulated vascular system of an angiosperm leaf. We evaluate the effect of leaf width-to-length proportion and orientation of second-order veins on the indexes of water transport into the leaves and sucrose transport from the leaves. We found that the most important factor affecting the steady-state pattern of hydraulic pressure distribution in the xylem and solute concentration in the phloem was leaf shape: narrower/longer leaves are less efficient in convecting xylem water and phloem solutes than wider/shorter leaves under all conditions studied. The degree of efficiency of transport is greatly influenced by the orientation of second-order veins relative to the main vein for all leaf proportions considered; the dependence is non-monotonic with efficiency maximized when the angle is approximately 45° to the main vein, although the angle of peak efficiency depends on other conditions. The sensitivity of transport efficiency to vein orientation increases with increasing vein conductivity. The vein angle at which efficiency is maximum tended to be smaller (relative to the main vein direction) in narrower leaves. The results may help to explain, or at least contribute to our understanding of, the evolution of parallel vein systems in monocot leaves.

## 1. Introduction

Vascular plants, particularly the dicot group of angiosperms, have evolved complicated hierarchical venation systems. The first-order so-called main vein of the system enters the leaf at the petiole and extends to the apex. Second-order veins diverge from this first-order vein. In turn, higher order veins branch off from their lower order neighbors to form a complex reticulated vein network extending across the entire leaf (Esau, 1953; Sack and Scoffoni, 2013). The detailed structure of the network differs notably from species to species, and potentially even from variety to variety within the same species. It is generally accepted that the whole-of-plant hydraulic system is significantly influenced by the hydraulic conductance of the plant's leaves (Sack and Holbrook, 2006). It follows then that leaf vasculature plays an important role in the response of a plant to water stress. In its evolution, however, a plant has also come to utilize this physical infrastructure to translocate to the rest of the plant, via a parallel phloem system connected to the petiole, photosynthetic products produced across the leaf (Roth-Nebelsick et al., 2001).

An underlying feature of note is that the network of xylem vessels runs parallel to the network of phloem vessels. Therefore, to properly appreciate the functionality of the vein architecture as a whole it is important to represent both the network's ability to conduct water from the petiole to the lamina and its capability to transport sucrose from the lamina to the petiole. This can only be achieved by allowing for the existence of both the phloem and the xylem sub-systems. In other words, it is important to represent both transport mechanisms as their respective roles perform in parallel. This is one of the objectives of our ongoing efforts. We note, however, that as the xylem and phloem networks do perform different functions it is equally interesting to identify conditions under which the two act independently, as it is to identify conditions under which the two influence each other, and naturally to understand the reasons for these opposing effects.

A large number of studies have reported on the relationship between leaf conductivity and vein architecture in conjunction with leaf shape. At a very basic level, leaf conductivity depends principally on vein density (Sack and Frole, 2006; Sommerville et al., 2012) and vein diameter (Cochard et al., 2004). But it has also been suggested that vein hierarchical architecture and vein tapering contribute to leaf conductivity (McKown et al., 2010). To what extent this is the case is still to be confirmed, but the number of connections among veins may be important (Brodersen et al., 2012). As for leaf shape, it is possible that the shape of lobed leaves confers the benefit of a high degree of conductivity by shortening the pathway toward sites of evaporation (Nicotra et al., 2011). Despite a high level of interest there has not been a systematic study of the effect of leaf shape or other important geometric characteristics, such as vein angle, on leaf water conductivity.

The majority of theoretical contributions to the study of plant vascular systems has focused on transport in stems or shoots. Early models considered xylem and phloem as isolated systems (Goeschl et al., 1976; Lhomme et al., 2001; Thompson and Holbrook, 2003; Steppe et al., 2006; Seki et al., 2015). More recent studies, however, explore the effects of the mutual influence of phloem and xylem in stems and roots of plants (Boersma et al., 1991; Daudet et al., 2002; Hölttä et al., 2006, 2009; Lacointe and Minchin, 2008; Foster and Miklavcic, 2013, 2014, 2016, 2017, 2019; Nikinmaa et al., 2013). These models confirmed the considerable influence of the phloem-xylem interaction on water transport (and in some cases solute transport). While some theoretical studies included a leaf component in a whole-of-plant transport system, no detailed description of leaf venation was featured; leaf transport properties were usually represented by a single parameter corresponding to a whole-of-leaf phloem and/or xylem conductance or resistance contribution.

Only a few studies focus attention on detailed modeling of water transport in leaves. But even these models considered the xylem as an isolated pathway, in a similar fashion to early stem models. Meinzer et al. (Meinzer and Grantz, 1990; Meinzer et al., 1992) used the well-known Ohm's law analogy to calculate sugarcane leaf conductance based on measurements of transpiration flow and water potentials. This analogy was utilized by subsequent authors (Zwieniecki et al., 2004, 2006). Xylem conductivity in another monocot plant, tall fescue *Festuca arundinacea* Schreb., was measured and compared to a theoretical estimate (Martre et al., 2001). The much more detailed model study of leaf vascular systems by Cochard et al. (2004) was then utilized by McKown et al. (2010) to study the impact of altering venation architecture traits. Measured pressure differences were used to model hydraulic architecture in dicot leaves of *Laurus nobilis* (Zwieniecki et al., 2002), while the pressure distribution in the xylem of a pine needle vein has also been modeled (Zwieniecki et al., 2006). North et al. (2013) developed a model for a monocot plant, tank bromeliad *Guzmania lingulata*, using leaky cable theory. What is common to all these studies is that the models employed only considered the xylem network.

In this paper we present and explore a detailed leaf model that features both the xylem and phloem networks. The xylem network model is the same as earlier models [particularly the model of Cochard, McKown and colleagues (Cochard et al., 2004; McKown et al., 2010)]. Our additional phloem network parallels the xylem network and is intimately coupled to the latter via additional conduits, as in an actual leaf; the phloem sap is modeled to flow according to well-defined hydraulic and osmotic pressure gradients in the phloem network. The coupled xylem-phloem network system we have adopted aims to imitate the hierarchical architecture of angiosperms.

The focus of this paper is on evaluating the effect of leaf shape and vein geometry on the functionality of both the xylem and the phloem networks. In our study, we consider, specifically, the effect of changing leaf shape (the length-to-width ratio), vein angle (and consequently vein density), and individual vein conductivities. We address the question of what is the resulting distribution of sucrose (a 2D leaf area concentration map) and the resultant hydraulic pressure pattern across the leaf, where the latter is used here as an index that characterizes the transport of both water and sucrose out of the leaf.

## 2. Method

### 2.1. Physical and Mathematical Model

**Figure 1:**
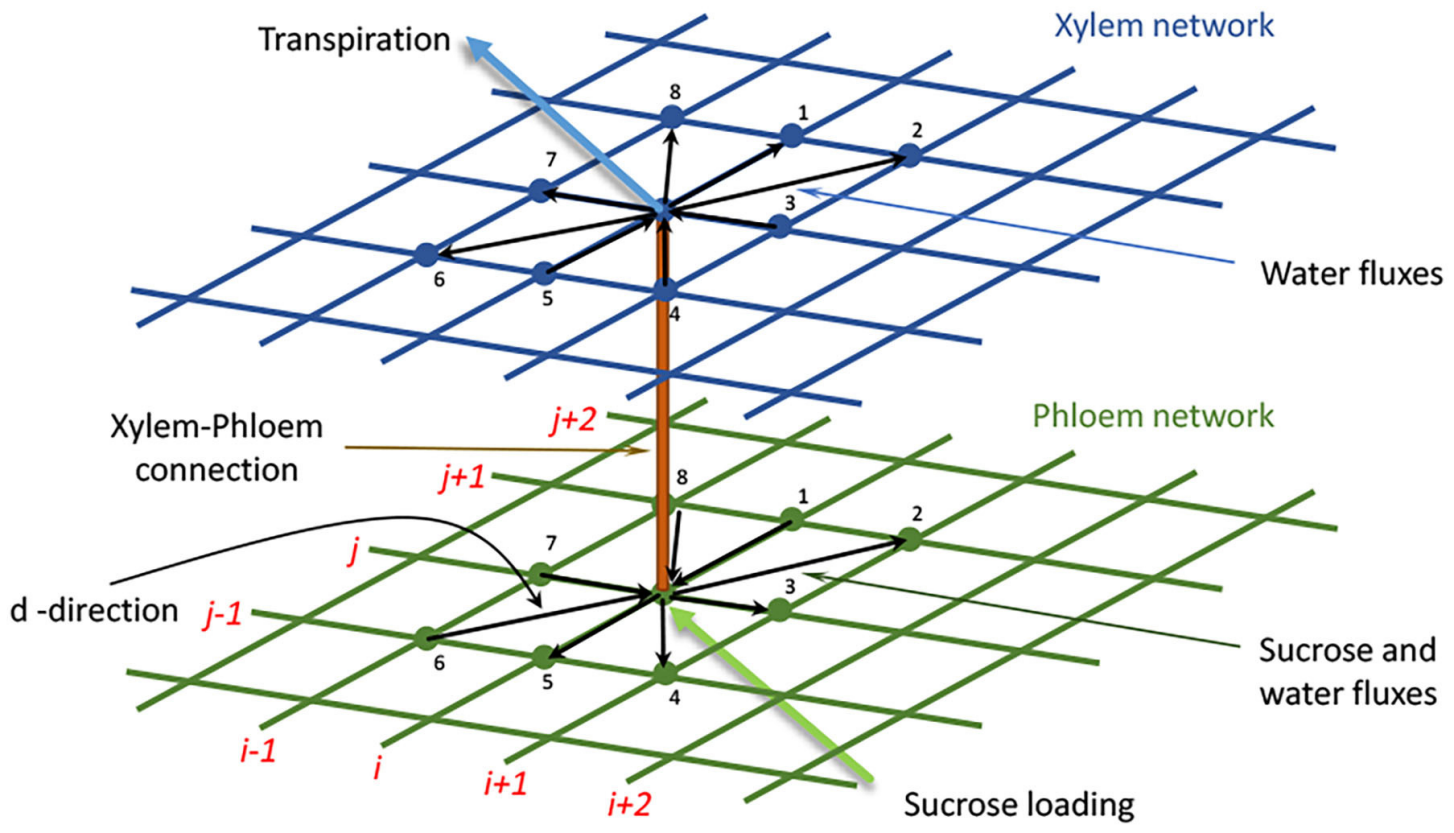
The xylem (upper blue) and phloem (lower green) networks, and their connections. The black arrows indicate the possible flow directions (d) to or from a single node to nearest and next nearest neighbor nodes within each network (these are numbered d = 1 to d = 8). The orange conduit connects a node in the xylem network to its corresponding partner node in the phloem network. The light blue arrow indicates transpiration *from* a xylem node, while the light green arrow indicates sucrose loading *to* a phloem node. In the xylem network, only water is transported, while in the phloem network both water and sucrose are transported. In our computations, the nodes are numbered in a grid-like fashion: (*i, j*) = (1, 1)…(*N, M*), as indicated by the red numbering. The identities of nearest and next-nearest neighbor nodes to node (*i, j*) are identified at the leaf creation stage and recorded for later recall.

The coupled xylem-phloem system we adopt is a significant extension of, but analogous to, the model developed by Cochard et al. (2004) and further utilized by McKown et al. (2010). In the original model the complex leaf vein architecture is presented as a two-dimensional network comprising xylem veins of different sizes (i.e., vein orders). The transport of fluid in the xylem and phloem systems is here modeled as a system of equations, founded on Darcy's law of plug flow (Batchelor, 1967): μ**u** = −*k*∇*p*, expressing the fact that in the conduit between two consecutive nodes the fluid velocity, **u**, is proportional to the pressure gradient across the conduit joining those nodes, with the vein conductance (*k*/μ) being the coefficient of proportionality; here *k* is a fluid permeability (m^2^) and μ is the fluid viscosity (Pa s), and the negative sign is consistent with flow from a point of high to a point of low hydraulic pressure. One of the main developments featured in our extended model is the addition of a parallel network of phloem veins, connected to the two-dimensional xylem network at corresponding nodes. In our model, every node (vein confluence) of the two-dimensional xylem system (blue points in the upper grid in Figure 1) is connected to a corresponding node in the phloem network (green points in the lower grid in Figure 1). In this phloem network we factored in the transport of sucrose solutes as well as water. The sucrose transport is influenced both by diffusive effects due to concentration differences between nodes, and by convective influences determined by hydraulic pressure differences between those nodes (see below).

At the (*i, j*)th xylem node (hereafter abbreviated to *ij*, for *i* = 1, …, *N, j* = 1, …, *M*), we apply the mass conservation constraint of zero net water flux (mmol s^−1^) out of the xylem node (i.e., total influx is balanced by total efflux). The conservation constraint asserts that the sum of the fluxes in the eight xylem conduit directions (see Table 1 and Figure 1), plus the flux from the xylem node to its corresponding phloem node, $F_{ij-c}^{xyl}$, and the transpiration flux, $F_{ij-T}^{xyl}$, is equal to zero.

(1) $$\begin{array}{l} \overset{8}{\underset{d=1}{\sum }}F_{ij-d}^{xyl}+F_{ij-T}^{xyl}+F_{ij-c}^{xyl}=0,\\ \;\left(i,j\right)=\left(1,1\right),\ldots ,\left(N,M\right). \end{array}$$

**Table 1:** Xylem flux directions (d).

**Number (d) **	**Direction **	**Variable name**
(1)	North	$F_{ij-1}^{xyl}$
(2)	North-east	$F_{ij-2}^{xyl}$
(3)	East	$F_{ij-3}^{xyl}$
(4)	South-east	$F_{ij-4}^{xyl}$
(5)	South	$F_{ij-5}^{xyl}$
(6)	South-west	$F_{ij-6}^{xyl}$
(7)	West	$F_{ij-7}^{xyl}$
(8)	North-west	$F_{ij-8}^{xyl}$

In the above equation, the transpiration flux ($F_{ij-T}^{xyl}$) is given as the product of a transpiration rate, *E*_*ij*_ (m s^−1^), and the 2D area per grid point (i.e., area per transpiring node), *a*_*ij*_ (m^2^): $F_{ij-T}^{xyl}=E_{ij}a_{ij}$ (Figure 1). In the original model of Cochard et al. (2004), a xylem node only possessed links with its four nearest neighbor nodes. In our extension, we also consider connections with next nearest neighbor xylem nodes, as indicated in the figure.

Arguing in a completely analogous fashion and therefore at a consistent level of approximation, we have, at the *ij*th phloem node, the zero sum of water fluxes in the eight lateral directions, $\left\{F_{ij-1}^{ph},\ldots ,F_{ij-8}^{ph}\right\}$, as well as the cross flow from the phloem node to its corresponding xylem node, $F_{ij-c}^{ph}$ ($=-F_{ij-c}^{xyl}$):

(2) $$\begin{array}{l} \overset{8}{\underset{d=1}{\sum }}F_{ij-d}^{ph}+F_{ij-c}^{ph}=0,\\ \;\left(i,j\right)=\left(1,1\right),\ldots ,\left(N,M\right). \end{array}$$

Invoking a discrete, cross-sectional area-integral version of Darcy's law (expressed in current nomenclature) the flux between two consecutive xylem nodes is determined from the relation

(3) $$\begin{array}{l} F_{ij-d}^{xyl}=\frac{k_{ij-d}^{xyl}A_{ij-d}^{xyl}}{\mu }\frac{\left(p_{d}^{xyl}-p_{ij}^{xyl}\right)}{l_{ij-d}^{xyl}},\\ \;=K_{ij-d}^{xyl}\left(p_{d}^{xyl}-p_{ij}^{xyl}\right),\\ \;\left(i,j\right)=\left(1,1\right),\ldots ,\left(N,M\right). \end{array}$$

In Equation (4), $F_{ij-d}^{xyl}$ is the (signed) water flux *from* xylem node *ij* *to* the xylem node connected to it in the direction *d*. Lastly,

(4) $$\begin{array}{l} K_{ij-d}^{xyl}=\frac{k_{ij-d}^{xyl}A_{ij-d}^{xyl}}{\mu {\;l}_{ij-d}^{xyl}}=L_{ij-d}^{xyl}A_{ij-d}^{xyl} \end{array}$$

is the conductance of the conduit between those nodes in terms of its cross-sectional area, *A*, length, *l*, permeability *k*, and fluid viscosity, μ. $L_{ij-d}^{xyl}$ is the local water permeability.

In the phloem, fluid motion is driven by hydraulic and osmotic pressure influences. Darcy's law must then be modified to include an osmotic pressure contribution resulting from a concentration difference:

$$\begin{array}{l} \Pi =-\gamma RT\Delta C, \end{array}$$

where *R* is the universal gas constant (8.314 J mol^−1^ K^−1^), *T* is temperature (in degrees K), and *C* is the local concentration (mol m^−3^) of solute (sucrose) inside the sieve tube. The parameter γ is a proportionality constant.

In discrete form, the cross-sectional area-integral of the flux in the phloem becomes

(5) $$\begin{array}{l} F_{ij-d}^{ph}=K_{ij-d}^{ph}(\left(p_{d}^{ph}-p_{ij}^{ph}\right)\\ \;-\sigma_{ij-d}RT(C_{d}^{ph}-C_{ij}^{ph})),\\ \;(i,j)=(1,1),\ldots ,(N,M). \end{array}$$

where, in direct analogy with the xylem case, $F_{ij-d}^{ph}$ is the (signed) fluid volume flux *from* phloem node *ij* *to* the phloem node connected to it in the direction *d* (one of eight neighbors). The position dependent parameter σ_*ij*−*d*_ is called the reflection coefficient (Katchalsky and Curran, 1965; Kramer and Boyer, 1995; Foster and Miklavcic, 2014, 2016). In Equation (6), σ_*ij*−*d*_ = 0 since the solute movement is assumed not to be impeded (Kramer and Boyer, 1995). $K_{ij-d}^{ph}=L_{ij-d}^{ph}A_{ij-d}^{ph}$ is the fluid conductance of the phloem conduit between the *ij*th node and its neighbor in the direction *d*, $L_{ij-d}^{ph}$ is its water permeability.

The (signed) volume flux of water *from* the xylem *to* the phloem, $F_{ij-c}^{xyl}$, (or vice versa) is determined by the difference between the hydraulic pressure at the xylem node and the sum of hydraulic pressure and osmotic pressure at the corresponding phloem node, multiplied by the conductance of the conduit linking those nodes. This is expressed by the relation:

(6) $$\begin{array}{l} F_{ij-c}^{xyl}=-F_{ij-c}^{ph}\\ \;=K_{ij-c}\left(\left(p_{ij}^{ph}-\sigma_{ij-c}RTC_{ij}^{ph}\right)-p_{ij}^{xyl}\right),\\ \;\left(i,j\right)=\left(1,1\right),\ldots ,\left(N,M\right). \end{array}$$

*K*_*ij*−*c*_ is the conductance of the conduit between the two nodes, defined as in Equation (4) and σ_*ij*−*c*_ is a reflection coefficient for this pathway, which is here set to unity (i.e., σ_*ij*−*c*_ = 1). From this we see that even if the hydraulic pressures in the xylem and phloem networks are equal, the presence of a solution in the phloem will drive fluid from the xylem to the phloem network.

We note in passing that at the scale of a typical leaf it is legitimate to ignore the influence of gravity when calculating water fluxes.

In direct analogy with the water fluxes, we assume a conservation of sucrose fluxes. Namely, we specify that the sum of all sucrose fluxes into and out from a given node in the eight lateral directions (*S*_*ij*−1_, ..., *S*_*ij*−8_), plus a contribution from sucrose loading into the sieve tube (*S*_*ij*−*L*_) should be equal to zero:

(7) $$\begin{array}{l} \overset{8}{\underset{d=1}{\sum }}S_{ij-d}+S_{ij-L}=0. \end{array}$$

In the above equation, the sucrose loading into the sieve tube (*S*_*ij*−*L*_) is calculated as *S*_*ij*−*L*_ = Λ_*ij*_*a*_*ij*_, where Λ_*ij*_ is the local sucrose loading rate per unit area and *a*_*ij*_ is the 2D grid area assigned to that node *ij*. We do not assume there to be any local depletion of sucrose due to consumption by metabolic processes, i.e., we assume no unloading of sucrose into leaf mesophyll tissue (although this may easily be added). On the other hand we do assume photosynthetic activity (sucrose production) at all nodal points and in all veins. Sucrose flow is driven by a combination of convection, which is proportional to the total pressure difference between neighboring phloem nodes, and diffusion, which depends on the sucrose concentration difference between those same two phloem vein nodes. This sum is expressed by the equation,

(8) $$\begin{array}{l} S_{ij-d}=\left(1-\sigma_{ij-d}\right)v_{\mu }F_{ij-d}^{ph}C_{ij}^{ph}\\ \;+D_{su}A_{ij-d}^{ph}\frac{C_{d}^{ph}-C_{ij}^{ph}}{l_{ij-d}},\\ \;=\left(1-\sigma_{ij-d}\right)v_{\mu }F_{ij-d}^{ph}C_{ij}^{ph}+G_{ij-d}^{ph}\left(C_{d}^{ph}-C_{ij}^{ph}\right),\\ \;i=1,\ldots ,N, \end{array}$$

where

$$G_{ij-d}^{ph}=D_{su}\frac{A_{ij-d}^{ph}}{l_{ij-d}}.$$

*S*_*ij*−*d*_ is defined as the mass flux (mol s^−1^) *from* node *ij* *to* the neighbor node in the *d* direction, $F_{ij-d}^{ph}$ is the corresponding volume flow of water, σ_*ij*−*d*_ = 0, and *D*_*su*_ is the free diffusion sucrose diffusivity (m^2^ s^−1^), and *v*_μ_ is the molal volume of water. All the symbols used in the model are summarized in Table 2.

**Table 2:** Summary of parameters and function variables (at node *ij*, subscripts not shown).

**Symbol**	**Description**	**Units**
*p*^*xyl*^	Xylem hydraulic pressure	MPa
*p*^*ph*^	Phloem total pressure	MPa
Π	Phloem osmotic pressure	MPa
*k*^*xyl*^	Xylem water permeability	m^2^
μ	Fluid (water) viscosity	Pa s
*C*^*ph*^	Phloem sucrose concentration	mol m^−3^
*F*^*xyl*^	Xylem water flux	mmol s^−1^
*F*^*ph*^	Phloem water flux	mmol s^−1^
*S*	Phloem sucrose flux	mol s^−1^
*K*^*xyl*^	Xylem conductance	mmol s^−1^ MPa^−1^
*K*^*ph*^	Phloem conductance	mmol s^−1^ MPa^−1^
*K*_*c*_	Phloem/xylem connection conductance	mmol s^−1^ MPa^−1^
$F_{T}^{xyl}$	Transpiration flux	mmol s^−1^
*S*_*L*_	Sucrose loading flux	mol s^−1^
*E*	Transpiration rate	mmol s^−1^ m^−2^
Λ	Sucrose loading rate	mol s^−1^ m^−2^
*a*	Area per grid point	m^2^
*A*^*ph*^	Phloem vein cross-sectional area	m^2^
*l*_−*d*_	Distance between nodes	m
*D*_*su*_	Sucrose diffusivity	m^2^ s^−1^
σ	Reflection coefficient	-
*v*_μ_	Molal volume of water	m^3^ mmol^−1^
*T*	Temperature of the leaf	K
*R*	Universal Gas constant	MPa m^3^ mol^−1^ K^−1^

### 2.2. Boundary Conditions

At every node, except the petiole, we adopt Dirichlet-type boundary conditions in which we assume fixed values of transpiration flow at all xylem nodes, and fixed sucrose loading at phloem nodes. By adopting Dirichlet boundary conditions, we essentially circumvent the need to calculate the water potential in the mesophyll wherein conductances in the narrow transpiration pathway between xylem conduits and mesophyll are uncertain. For the transpiration rate, *E*_*ij*_, we set (for comparative reasons) a baseline or reference value of −2.00 mmol s^−1^ m^−2^, although in our exploration we also consider lower values. For the sucrose loading rate, Λ_*ij*_, we assigned a reference value of 2.78 × 10^−7^ mol s^−1^ m^−2^, although in this case too we consider lower values. With this choice of parameters we arbitrarily assume that approximately one-third of all photosynthate production is loaded into phloem sieves given an ordinal leaf photosynthesis production of 1.00 × 10^−6^ mol (CO_2_) s^−1^ m^−2^.

At the petiole, the hydraulic pressure in the xylem we arbitrarily set to 0.00 MPa, while the pressure in the phloem was set to 0.20 MPa. With regard to sucrose transport, we adopt a zero Neumann boundary condition at the petiole, which is equivalent to stating that the sucrose concentration at the petiole is equal to the sucrose concentration at the exterior phloem “node” connected immediately to it.

The choices of these types of boundary conditions, as well as the actual values set, are purely for pragmatic reasons as we wish to explore the effects of other variables on water and solute transport. In subsequent studies we shall adopt more physically relevant conditions as we move from the present steady-state model to a time-dependent model.

### 2.3. Leaf and Vein Structure

**Figure 2:**
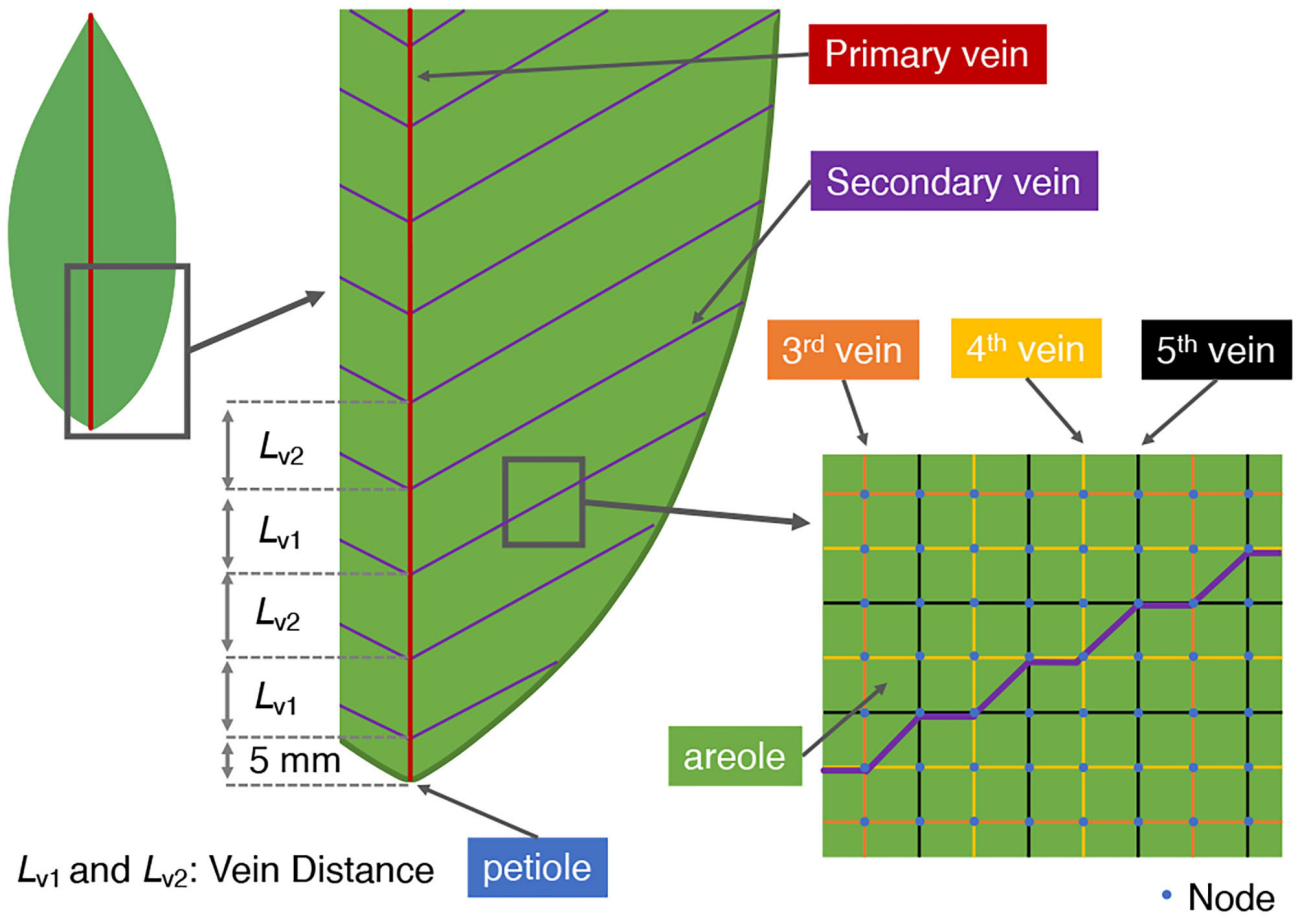
Vein architecture of the model leaf. In our simulations, the angle of the second-order vein to the main vein is varied. Accordingly, the vein distances *L*_*v*1_ and *L*_*v*2_ are also varied (alternately) to ensure a constant total vein length.

In the present paper we shall often refer to so-called baseline or reference models of leaf shape and vein architecture. These correspond to the Laurel leaf shape and vein structure adopted and studied in the work of Cochard et al. (2004). The first-order vein is marked by the red line in Figure 2, and depicts the main vein connected directly to the petiole at the base of the leaf. The second-order veins shown in purple in Figure 2, branch directly off from the first-order vein at regular intervals and at a finite, acute angle, as measured from the main vein. Higher order veins (third, fourth, and fifth order veins) are arranged on and aligned with a rectangular lattice of nodal points (blue dots in Figure 2). They are distinguished by their frequency of occurrence as well as by their cross-sectional areas and permeabilities. The third-order veins (marked by orange lines in Figure 2) are distributed, both vertically and horizontally, at a frequency of every six nodal points. The two narrowest veins, the fourth and fifth order veins, marked by yellow and black lines, respectively in Figure 2, occur at the same frequency, but with their respective networks displaced by one rectangular grid unit so as to appear alternately in a similar rectangular pattern. On the main vein, which is the leaf's symmetry axis, the internal node which is connected directly to the petiole is set a distance of 5 mm from the petiole.

In our study we considered the effect on the xylem hydraulic pressure, the phloem pressure, and the phloem distribution of sucrose, of varying the angle of the second-order veins relative to the main vein direction. In order to make a reasonable comparison of quantities under different simulation conditions the total lengths of the veins of all orders were kept fixed. Two factors need considering in implementing this vein length constraint.

First, increasing the angle θ of the second-order veins reduces their lengths, since the distance from the main vein to the edge of the leaf along a second-order vein becomes shorter. Therefore, to maintain the total length of second-order veins at all angles we reduced the interval between the second-order veins and increased their number as the angle of the veins increased. As a reference state we assign the case of θ = 0° where the second-order veins are perpendicular to the first-order vein; the vein spacing was then set at 10.00 mm, while the distance between the very first second-order vein and the petiole was set at 5.00 mm (Figure 2). The intervals, *L*_*v*1_ and *L*_*v*2_, between connection points of alternate second-order veins (shown in Figure 2) were then adjusted (to different extents) in order to keep constant the total length of second-order veins. The total length of second-order veins was set at 0.74 ± 0.01 m S.D.).

### 2.4. Parameter Settings

**Figure 3:**
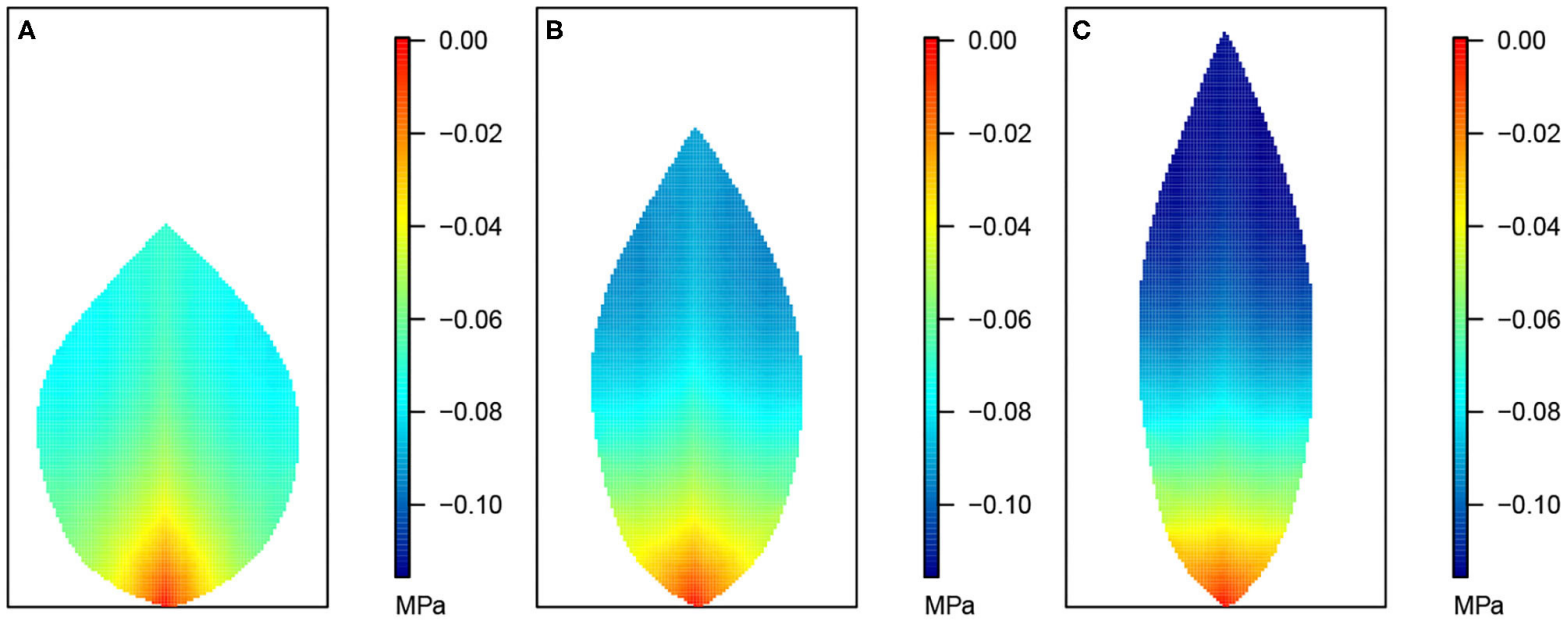
Leaf area distributions of xylem hydraulic pressure. In this and the next three figures, **(A)** depicts the wide leaf, **(B)** shows the reference leaf, while **(C)** shows the narrow leaf distribution. In addition, second-order veins are 45° to the main vein (i.e., 45° to the reference state of perpendicular veins). Parameter conditions under which the simulations were performed are summarized in Table 3 and the core values in Table 4.

**Figure 4:**
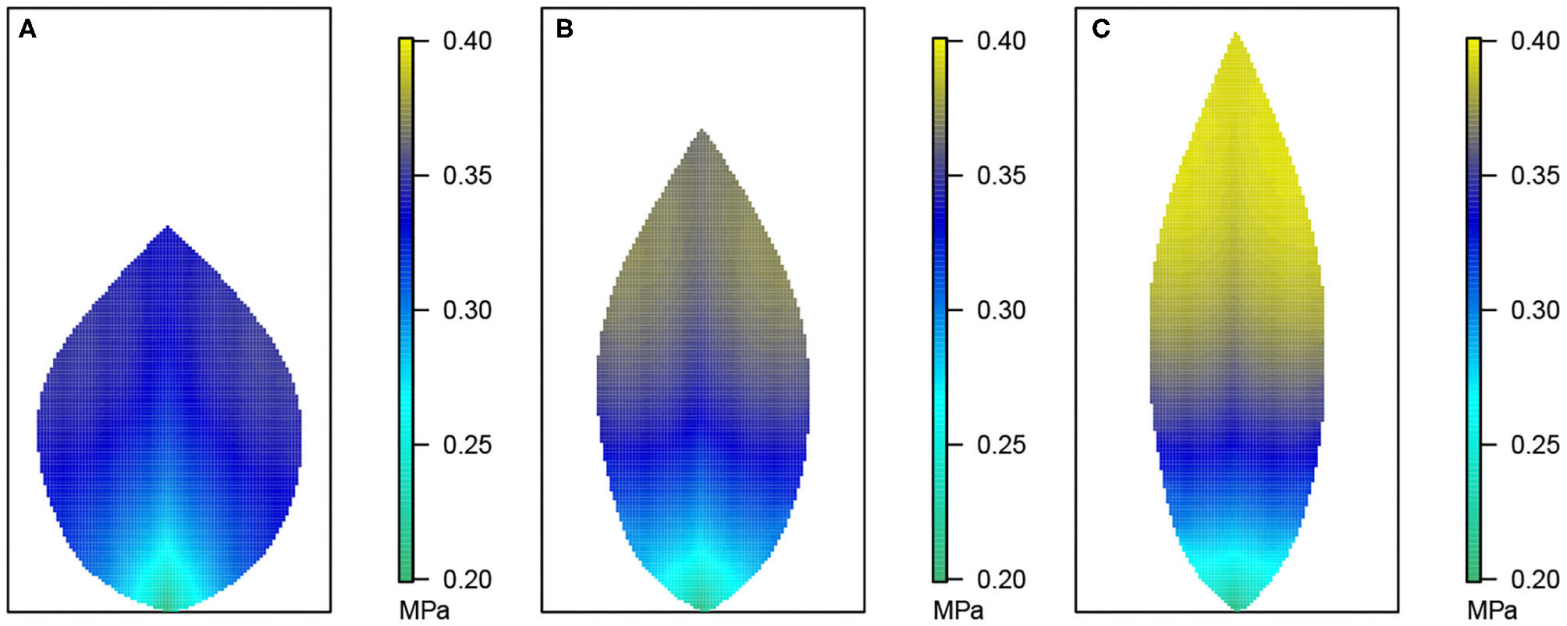
Leaf area distribution of hydraulic pressure in the phloem network. **(A)** Wide leaf, **(B)** reference leaf, and **(C)** narrow leaf. Details as in Figure 3.

**Figure 5:**
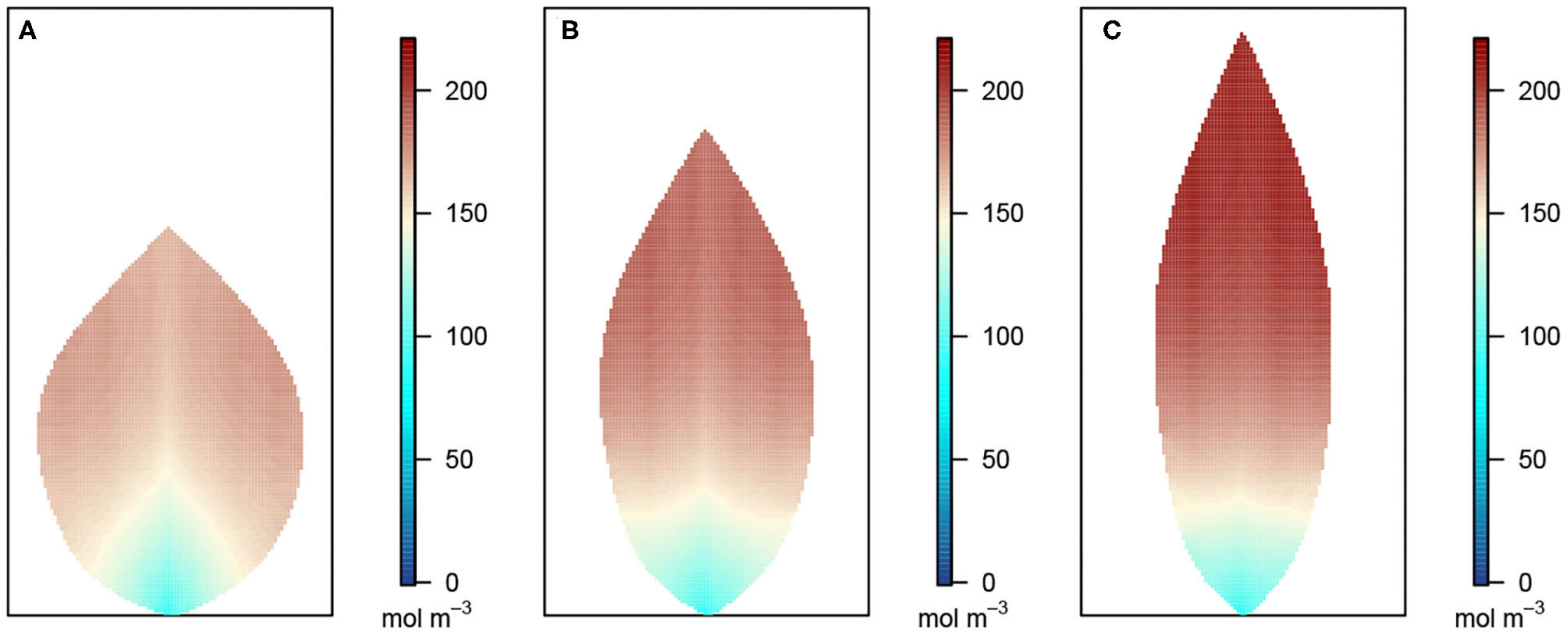
Leaf area distribution of sucrose concentration in the phloem network. **(A)** Wide leaf, **(B)** reference leaf, and **(C)** narrow leaf. Details as in Figure 3.

The main aim of the study was to evaluate the effects of changing leaf shape and changing vein angle on the indices that reflect water flow into and through leaves, as well as on the sucrose flow from leaves. We designed three (virtual) leaf shapes that differ in their length-to-width ratio. These are illustrated in Figures 3–5. As mentioned earlier, the reference leaf shape with which we compare other shapes and vein architectures is that of the Laurel leaf described in Cochard et al. (2004). For this leaf we have assumed a leaf length of 16.00 cm and maximum leaf width of 6.47 cm. The other cases we consider are either wider (8.09 cm) or narrower (5.39 cm) leaves, with leaf lengths being, respectively, 80 and 120% of the length of the reference leaf shape. In all three cases the leaf areas are maintained at 73.35 cm^2^. For each leaf shape, our simulations were conducted assuming the following 2^nd^ order vein angles (differing from each other by approximately 6°): 0.00°, 7.13°, 14.03°, 21.80°, 30.96°, 36.87°, 40.60°, 45.00°, 49.40°, 53.13°, 59.06°, 68.20°, and 75.96°. The irregular increments are due to the need to adapt angles to our discrete grid. The vein angle of θ = 0° corresponds to our reference state of perpendicular 2^nd^ order veins.

As for the choice of values of individual vein conductances, we defer to the values adopted in (Cochard et al., 2004). Consequently, the 5^*th*^ order veins have the lowest conductance values, with the conductances increasing with decreasing vein order in correspondence with the envisaged increases in vein diameter. The specific reference values are tabulated in Table 3.

**Table 3:** Xylem vein conductivities; phloem conductivities are a factor of 3/100 lower.

**Vein order **	**Conductance **	**Vein diameter**
** **	**(m mmol s^**−1**^ MPa^**−1**^) **	**(μm)**
1	1.00 × 10^−2^	15.50
2	5.00 × 10^−4^	11.30
3	6.00 × 10^−5^	7.50
4	4.00 × 10^−5^	7.20
5	4.00 × 10^−6^	4.34

Apart from studying the effect of leaf shape and vein angle we also consider the influence of 2^nd^ order vein conductance. To this end we performed simulations with 2^nd^ order vein conductances that were 5 times larger and 5 times smaller than the reference value given in Table 3.

It is important to point out here that, as suggested by Daudet et al. (2002), we have assigned values for the conductances in the phloem network that were smaller than the conductances of corresponding veins in the xylem by a uniform factor of 3/100 (Not being the focus of this paper, with this single choice of relation between the two networks it is not possible to draw any significant conclusions on the interaction between the two networks).

A uniform value of conductance for the conduits between the phloem and xylem networks was set at 0.50 mmol s^−1^ MPa^−1^ (Daudet et al., 2002). Other fixed parameters are the gas constant as *R* = 8.31 × 10^−6^ MPa m^3^ mol^−1^ K^−1^, sucrose diffusion coefficient as $D_{su}=5.22\times 10^{-10}$ m^2^ s^−1^, water volume per mmol as $v_{\mu }=18.00\times 10^{-9}$ m^3^ mmol^−1^, and leaf temperature as *T* = 293.00 K.

Finally, under the reference simulation condition, the sucrose loading rate in the phloem network was set initially to be uniform across the grid. However, given that leaf conductance, hydraulic pressure, and sucrose concentration may vary if the loading rate is not uniform, we considered also a case of non-uniform sucrose loading profile: a linear gradient starting with a 50% higher loading rate at the leaf base than the average, linearly decreasing to a 50% lower loading rate at the leaf tip. The average loading rate in both cases was kept the same. The summary of the parameter values of the base experimental setting and optional settings are shown in Table 4.

**Table 4:** Summary of system parameters with core and additional values.

**Parameter**	**Unit**	**Core model value**	**Altered value(s)**
Inter-vein interval (*L*_*v*1_; *L*_*v*2_)	(mm)	10.00	15.00
Sucrose loading gradient (∂*S*_*ij*−*L*_/∂*z*)	(%)	0.00 (uniform)	1.0 (linear gradient)
Uniform transpiration rate (*E*)	(mmol s^−1^ m^−2^)	-2.00	-1.00; 0.00
Phloem/xylem conduit conductance (*L*_*ij*−*c*_)	(mmol s^−1^ MPa^−1^)	0.5	0.0005
Second-order vein conductance (*K*^*xyl*^ = *K*^*ph*^)	(m mmol s^−1^ MPa^−1^)	5.00 × 10^−4^	1.00 × 10^−4^; 2.50 × 10^−3^

In this work, we present the majority of our results in terms of the average leaf xylem hydraulic pressure, defined as

(9) $$\begin{array}{l} \langle p_{leaf}^{xyl}\rangle =\frac{1}{NM}\sum_{i=1}^{N}\sum_{j=1}^{N}p_{ij}^{xyl}. \end{array}$$

An analogous expression applies to the average phloem pressure, $\langle p_{leaf}^{ph}\rangle$.

By solving Equations (2), (3), and (7), we determined values for $p_{ij}^{xyl}$, $p_{ij}^{ph}$, and *C*_*ij*_ for each node. Equations (2), (3), and (7) were solved numerically using the Matlab (Mathworks, 2020) non-linear system solver *fsolve*, with the default setting employing the Trust-Region Dogleg Method.

## 3. Results

### 3.1. Pressure and Solute Distribution Patterns

Figure 3 shows the 2D distribution of the xylem hydraulic pressure for our three leaf shapes: the wide leaf (Figure 3A), the reference leaf (Figure 3B), and the narrow leaf (Figure 3C), assuming that 2^nd^ order veins are 45° to the main vein (i.e., 45° to the reference state of perpendicular veins). Although the boundary conditions and total vein lengths were identical in these three cases, the pressure distributions were different. While all three cases showed similar qualitative behavior: the xylem hydraulic pressure was lower at the leaf tip and higher closer to the petiole, the rate of decrease of hydraulic pressure (from the petiole to the leaf tip) was greater for narrower leaves. In contrast, Figure 4 shows the distribution of hydraulic pressure in the phloem network. An inverse correspondence to that of the xylem hydraulic pressure distribution is apparent, the phloem pressure increased from the leaf petiole to the leaf tip. Once again we found that the narrower was the leaf, the steeper was the gradient. On the other hand, the area-average phloem pressure in the three leaves varied only marginally from 0.33 MPa for the wide leaf, to 0.34, and 0.36 MPa, respectively, for the reference leaf and the narrow leaf.

The data in Figures 3, 4 shows that while water in the xylem flowed from the petiole out toward the extremities, water in the phloem flowed in the opposite direction, from the extremities to the petiole. This suggests there was a degree of circulation between the two networks, on a length scale of the whole leaf.

Figure 5 shows the sucrose concentration profiles in the three leaf shapes. The predicted sucrose concentration pattern mimicked that of the pressure pattern in the phloem: higher at the extremities and lower at the petiole. However, the solute concentration gradient appears somewhat different in shape compared with the pressure gradient suggested in Figure 4, with the concentration being more uniform over a larger part of the leaf before decreasing very rapidly over the one-third leaf section closest to the petiole. This pattern is reflected also in the larger differences between the area-average sucrose concentrations found for the three leaf shapes: 159.30, 169.37, and 181.82 mol m^−3^ for the widest, the reference and the narrowest leaf, respectively.

### 3.2. Effect of Vein Angle and Leaf Shape

**Figure 6:**
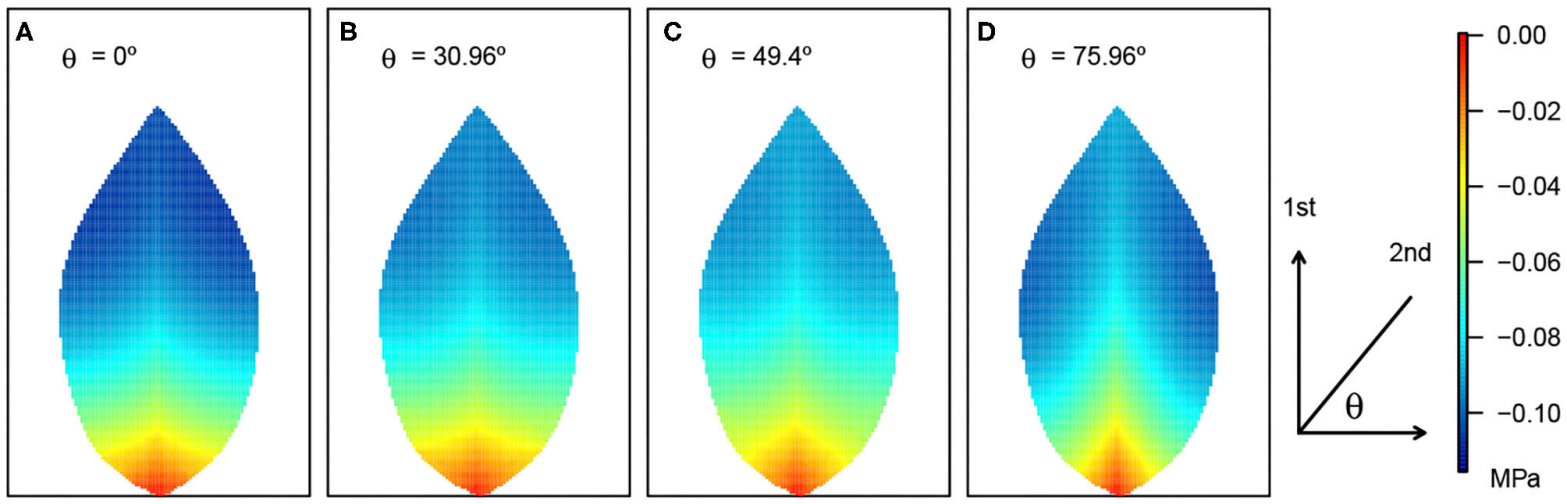
Leaf area distribution of xylem hydraulic pressure and its response to varying angle of second-order veins. The resulting patterns for four θ angles are shown in **(A–D)**. The definition of the angle θ is shown adjacent to **(D)**. The pressure scale is given on the right. Other parameter details are as in Figure 3.

**Figure 7:**
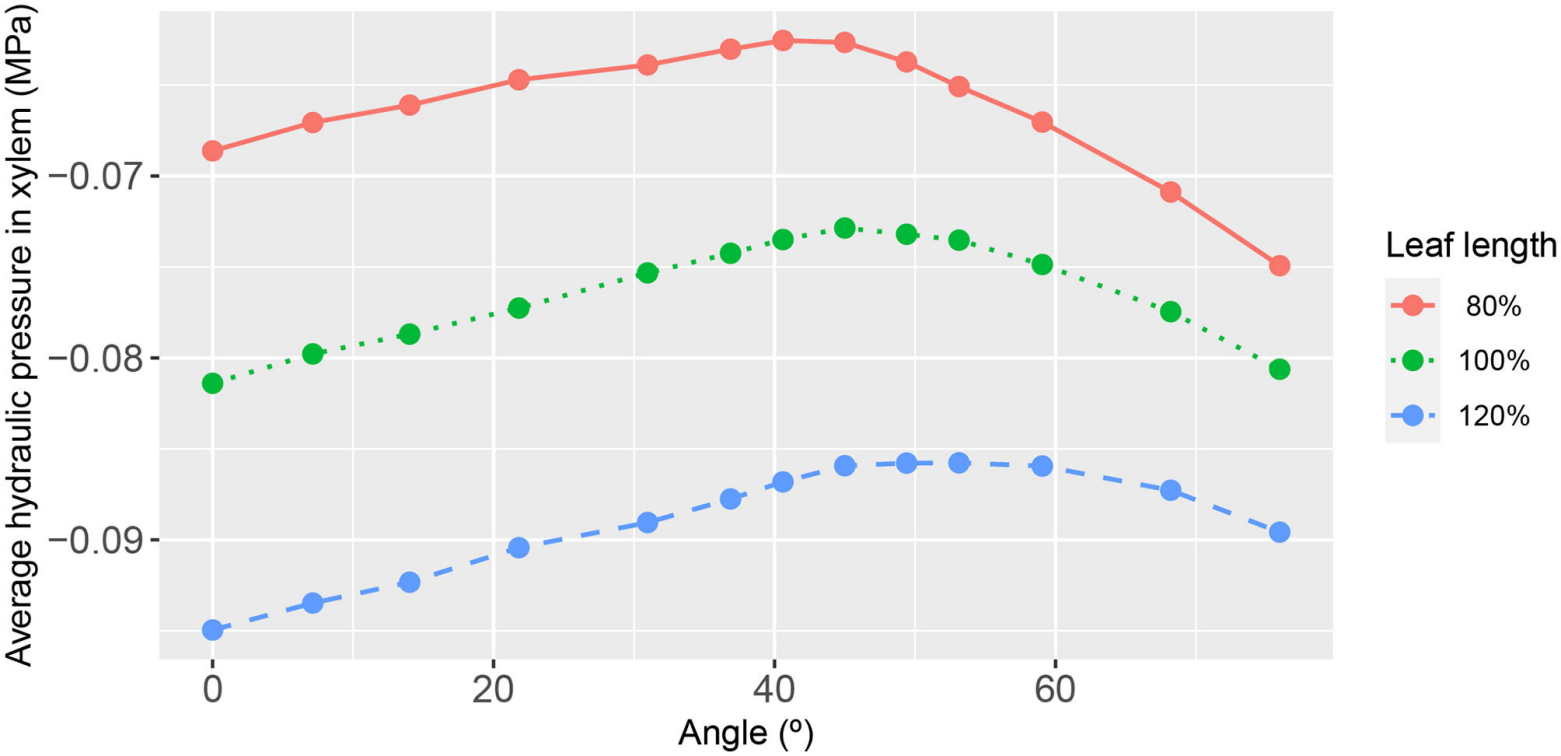
The functional relationship between leaf area-average, xylem hydraulic pressure and second-order vein angle. The different curves refer to the narrower/longer leaf (blue dashed line), the reference leaf (green dotted line), and the wider/shorter leaf (red solid line). The parameter values for these results are as in Figure 3.

**Figure 8:**
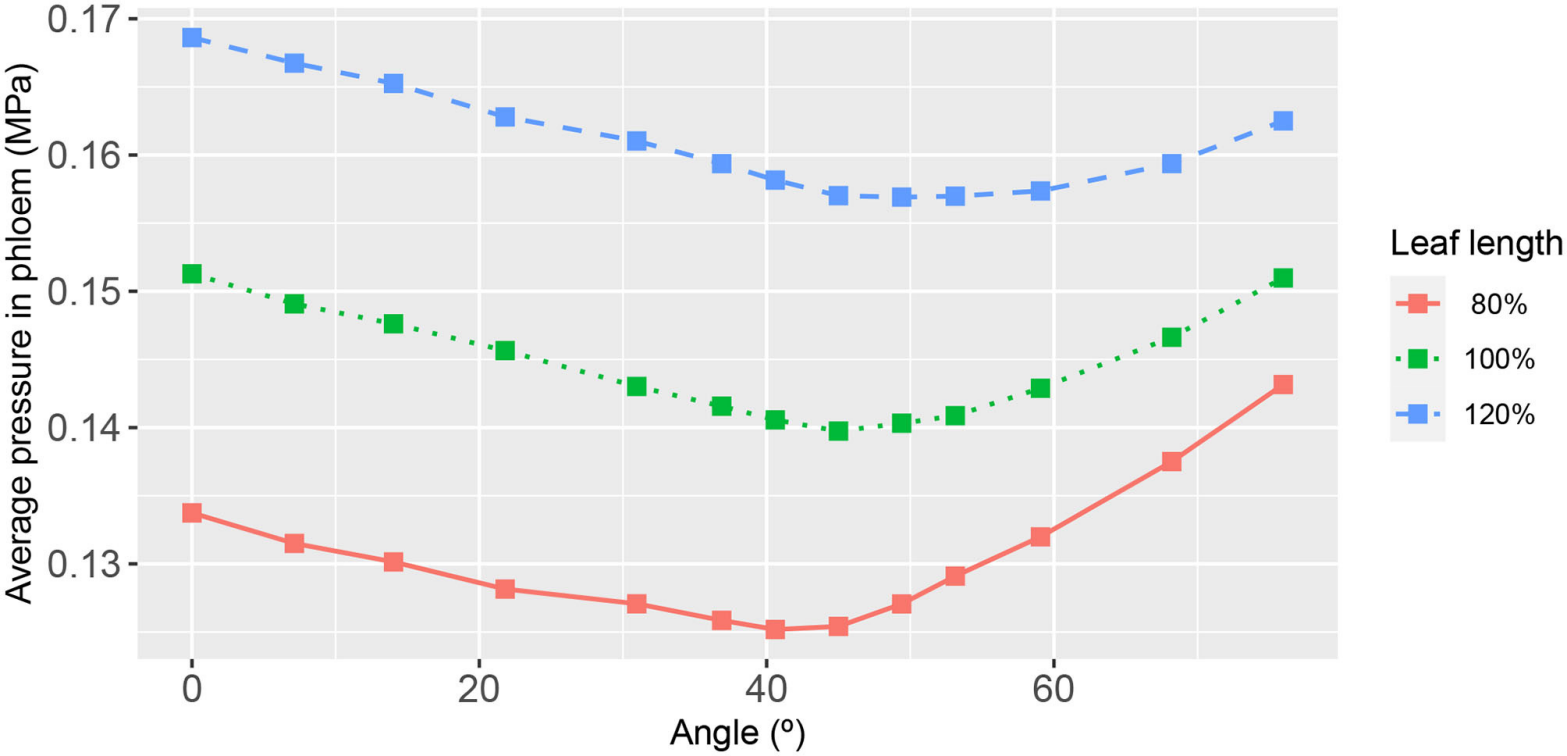
The functional relationship between leaf area-average, hydraulic pressure in the phloem, and second-order vein angle. Curve descriptions and simulation conditions are as in Figure 3.

In this section, we compare the effect of varying the angle of second-order veins (with respect to the main vein) on the xylem hydraulic pressure, phloem hydraulic pressure and sucrose concentration. As mentioned earlier the reference state (θ = 0) has 2^nd^ order veins being perpendicular to the main vein. Figure 6 shows the effect on the distribution of hydraulic pressure in the xylem for a leaf of a given shape (the reference shape) as a result of varying the angle of orientation of second-order veins. Note that increasing θ (from θ = 0 corresponding to perpendicular 2^nd^ order veins) means veins become more aligned with the main vein. Although 13 different θ angles were sampled, only four case distributions are shown here. All cases are summarized in Figures 7, 8, where we have plotted area-average quantities against vein angle.

While the quantitative differences in the area-average xylem hydraulic pressure, over the angle range studied, were not great, it is nevertheless significant that the effect of a monotonic decrease in 2^nd^ order vein angle was a non-monotonic response. This non-monotonic response itself appeared to be non-uniform across the leaf as can be observed in Figure 6, where close to the petiole and close to the main vein, the response appeared to be directly proportional. Elsewhere, e.g., toward the leaf edge, the effect was clearly non-monotonic. Given that the area-averages in Figures 7, 8 were themselves non-monotonic, it would appear that the observed non-uniform effect away from the symmetry line dominated.

The summary relationships between second-order vein angle and leaf area-average xylem hydraulic pressure is shown in Figure 7. In concert with the specific case of 45° aligned 2^nd^ order veins (shown in Figures 3–5), the area-averaged hydraulic pressure was generally lower the narrower was the leaf, at all angles. We also noted that the peak in the average pressure was around 45°, although the angle at which the peak was reached was not constant (varying from 40.60° for the widest leaf to 53.13° for the narrowest leaf). Moreover, it is distinctive that the shapes of the curves were not symmetric about the peak position, with a steeper decay in average hydraulic pressure for larger angles (i.e., for veins more acutely aligned with the main vein).

As may be expected, the response of the leaf area-averaged phloem hydraulic pressure (Figure 8) was approximately the inverse of that of the xylem hydraulic pressure. The average phloem pressure was higher in the narrowest leaf, at all angles of 2^nd^ order veins. This is consistent with the earlier finding that the solute concentration was higher generally in the narrowest leaf. Interpreting the pressure as a potential for transporting photosynthate solutes out of the leaf via the phloem (by diffusion), the results suggest that narrow leaves have (and probably need) a greater potential to convey these solutes than do wider leaves. Moreover, leaves attain a still higher pressure by increasing or decreasing the angle of their 2^nd^ order veins relative to the roughly 45° orientation.

### 3.3. Effect of Vein Angle and Leaf Shape Under Other Conditions

Steady state simulations investigating the effect of 2^nd^ order vein angle on the leaf hydraulics were also performed under other external condition settings (see the final column in Table 4). These included different pressure boundary condition at the petiole, evaporation rates, *E*, sucrose loading rate, *S*_*ij*−*L*_, and vein conductances. As these results appear qualitatively similar to the results shown above, we simply comment here on the simulation outcomes and relegate the figures to the Supplementary Material.

With regard to the influence of transpiration rate, we considered rates of transpiration reduced (in magnitude) from our reference value of −2.00 mmol s^−1^ m^−2^ to *E* = −1.00 and to *E* = 0.00 mmol s^−1^ m^−2^ (i.e., no transpiration). Even under the extreme condition of no transpiration, the results of leaf area-average xylem hydraulic pressure and area-average phloem total pressure were qualitatively the same as found with a transpiration setting of *E* = −2.00 mmol s^−1^ m^−2^) (see Supplementary Figures 1–3). This suggests that the nature of the response is intrinsic to the leaf itself and not a consequence of transpiration.

Adjusting the pressure boundary condition from *P* = 0.0 MPa to *P* = −2.0 MPa, while maintaining the same difference in pressure between xylem and phloem at the petiole (so the phloem pressure was then −1.8 MPa) resulted only in a magnitude change in the average xylem pressure but no relative difference in either the trend with 2^nd^ order vein angle or the relation between the different leaf shapes (Supplementary Figure 4).

Thirdly, as another external condition that might influence the behavior of the pressure distributions, we considered a non-uniform (linear gradient) sucrose loading rate, ranging from 50% higher than the average at the leaf base to 50% lower at the leaf tip. Although the distribution pattern of sucrose concentration with this uneven sucrose loading was different from that found with a uniform distribution (see Supplementary Figure 5), the relationships between the leaf area-average xylem hydraulic and phloem hydraulic pressure, the vein angle (or leaf shape) were similar to those found with a uniform sucrose loading (Supplementary Figures 6, 7).

**Figure 9:**
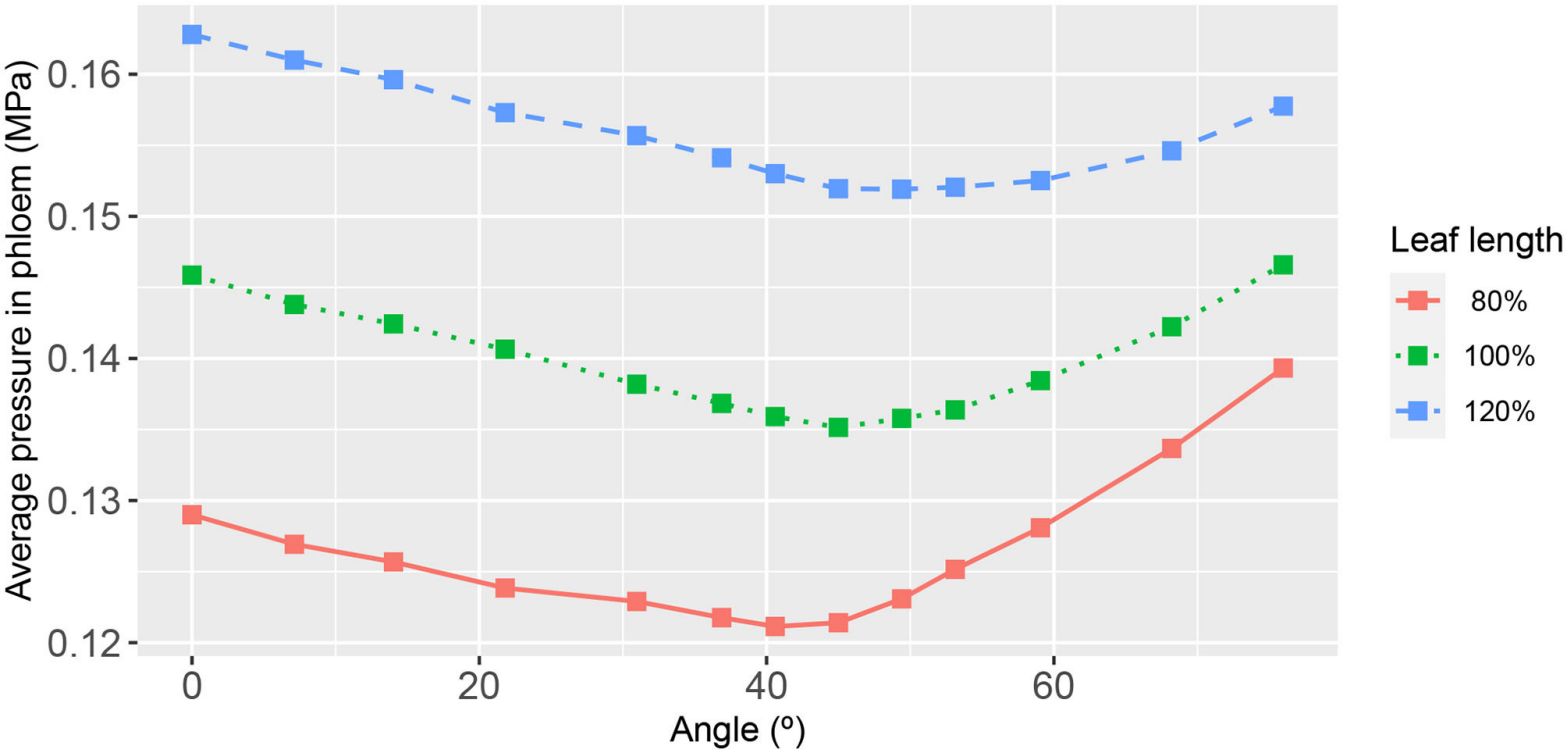
The functional relationship between leaf area-average, hydraulic pressure in the phloem and second-order vein angle. Curve descriptions and simulation conditions are as in this figure except with a xylem/phloem conduit conductance of $K_{ij-c}=5\times 10^{-4}$ mmol s^−1^ m^−2^ (i.e., reduced by a factor of 10^3^).

Since previous whole-leaf models treated only the xylem network (Meinzer and Grantz, 1990; Meinzer et al., 1992; Cochard et al., 2004; Zwieniecki et al., 2004, 2006; McKown et al., 2010) we thought, in our fourth exercise, to see whether the hydraulic response in the leaf xylem to a variation of 2^nd^ order vein angle was influenced by the connection to the phloem network. We considered one case where the conductance of the phloem/xylem conduit connection was significantly reduced (by three orders of magnitude) to virtually isolate the xylem and phloem networks. Interestingly, the leaf area-average xylem hydraulic pressures were largely unaltered (Supplementary Figure 8). As for the leaf area-average phloem pressure, although the magnitudes were lower than under a higher xylem/phloem conductance, the resulting dependencies on 2^nd^ order vein angle (or leaf shape) were qualitatively similar (Figure 9). It is a little premature to draw any general conclusion from this single result as the outcome may be a consequence of the particular set of all parameters.

To address the question of whether the higher order veins play a critical role in the xylem hydraulic pressure distribution, in our fifth effort we repeated our simulations for the two cases of either both 4th and 5th order veins having the higher of the two original conductivities (Supplementary Figures 9, 10), or both having the lower of the two original conductivities (Supplementary Figures 11, 12). Although the magnitudes were (again) different in either case, the qualitative behavior of the leaf area-average xylem hydraulic pressure to 2^nd^ order vein angle (and leaf shape) were similar. The leaf area-average phloem hydraulic pressure followed suit. Somewhat unsurprisingly, increasing the conductances of the higher order veins, such as fifth order veins (Supplementary Figure 9), demonstrates a weakening of the influence of the 2^nd^ order veins. As higher order veins become more conductive the dependence of the 2^nd^ order vein angle becomes less pronounced since the distribution of water (and solutes) is more equally shared between 2^nd^ order and higher order veins. As could be expected, by lowering the conductivities of higher order veins (relative to 2^nd^ order veins) the effect of 2^nd^ order vein angle (Supplementary Figure 11) was enhanced.

**Figure 10:**
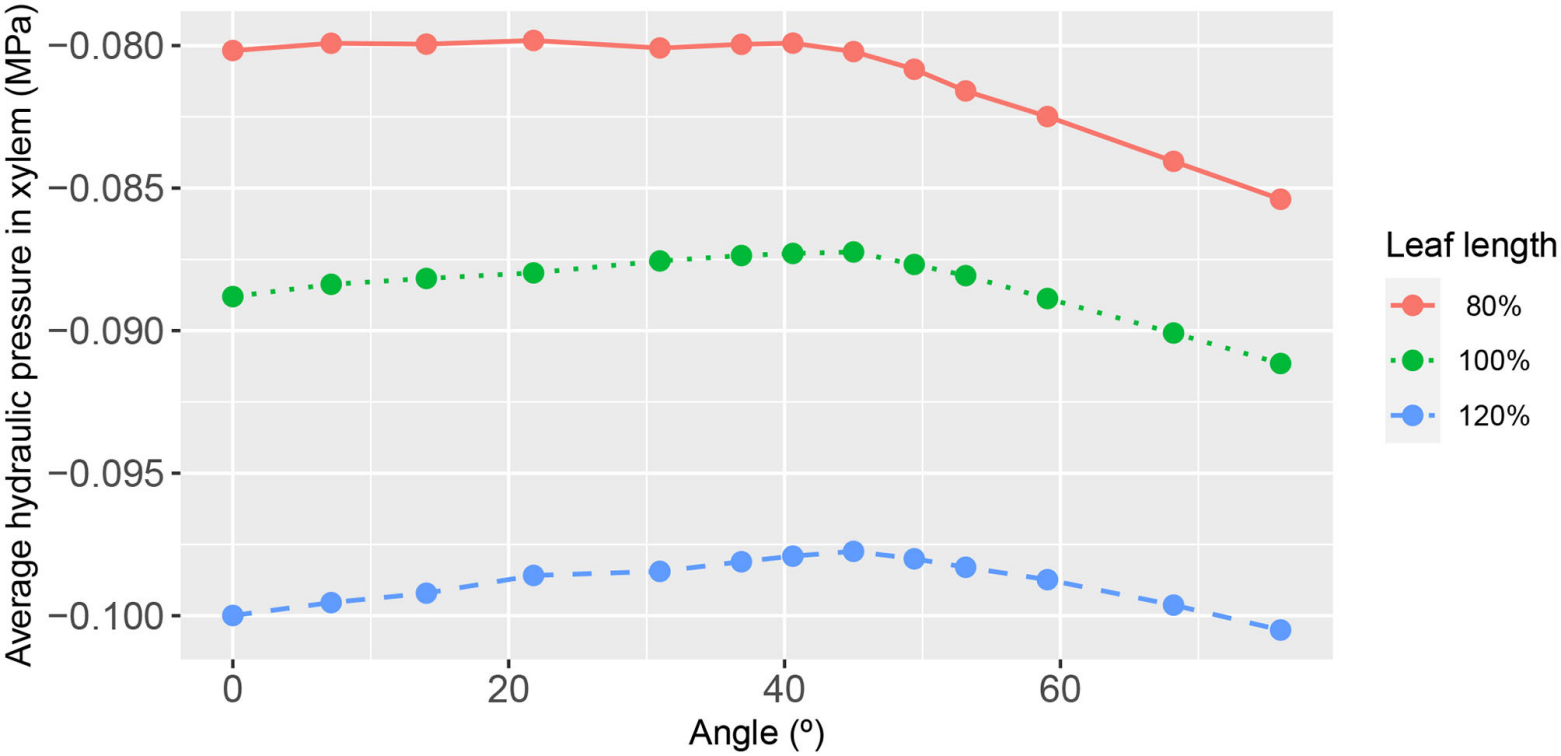
The functional relationship between leaf area-average, xylem hydraulic pressure, and second-order vein angle. Curve descriptions and simulation conditions are as in Figure 7 except with a uniform second-order vein conductance of *K*^*xyl*^ = 100*K*^*ph*^/3 = 1 × 10^−4^ mmol s^−1^ m^−2^ (i.e., a factor of one-fifth that of the core value).

Somewhat on the same theme, we evaluated the leaf response for different conductances of the 2^nd^ order veins themselves. Figure 10 shows the dependence on 2^nd^ order vein angle of the leaf area-average xylem hydraulic pressure for a reduced 2^nd^ order vein conductance of one-fifth that of the core model setting. While the overall magnitudes were very low, it is nevertheless distinguishable in that, within a class of leaf shape, the response to a change of angle was weakened: the degree of variation was 2, 1.3, and 0.9% for the wide leaf, the reference leaf and the narrow leaf, respectively. This result was also consistent with the diminished role of 2^nd^ order veins when the conductances of the higher order veins were increased relatively speaking (Supplementary Figures 9, 10). As previously, the phloem hydraulic pressure shows an inverted response (see Supplementary Figure 13).

**Figure 11:**
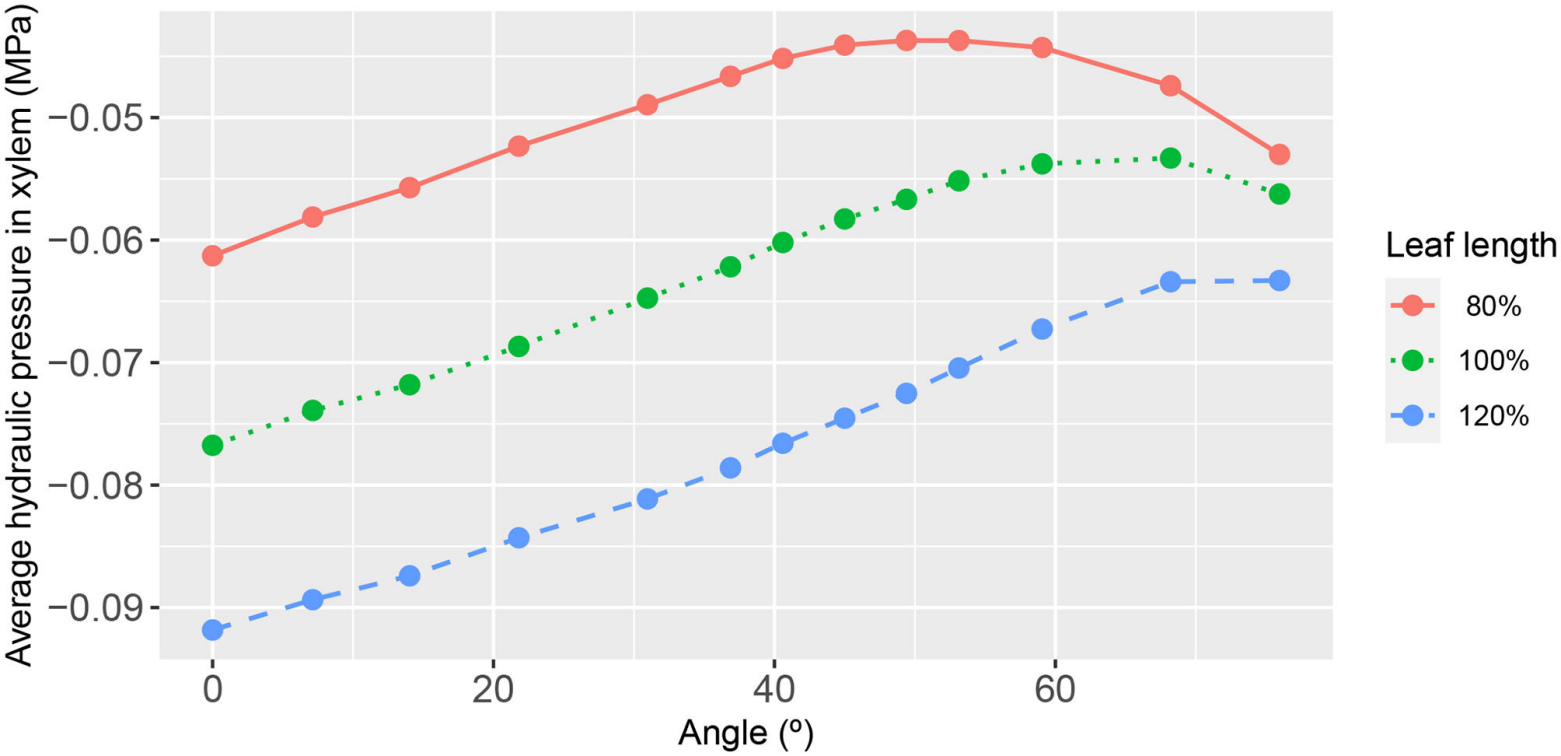
The functional relationship between leaf area-average, xylem hydraulic pressure, and second-order vein angle. Curve descriptions and simulation conditions are as in Figure 7 except with a uniform second-order vein conductance of *K*^*xyl*^ = 100*K*^*ph*^/3 = 2.5 × 10^−3^ mmol s^−1^ m^−2^ (i.e., an increase by a factor of 5 of the core value).

Not surprisingly, in the contrasting case of an increase in 2^nd^ order vein conductance, the dependence on vein orientation was enhanced, as shown in Figure 11 for which the conductance of second-order veins was increased by a factor of 5. The estimated degrees to which the average xylem pressure changed with angle were 11, 12, and 13% for the wide leaf, the reference leaf, and the narrow leaf, respectively. The angle at which the xylem pressure peaked changed significantly: 49°, 68°, and 76°. Again the angular dependence of leaf area-average phloem pressure showed an inverted relationship to that of the leaf area-average xylem hydraulic pressure (see Supplementary Figure 14).

## 4. Discussion

This study of a coupled xylem/phloem model of an angiosperm leaf with a reticulated vein network focused on the effect of specific geometrical features (leaf shape and second-order vein angle) on the hydraulic capability of the interconnected xylem and phloem system. The indices we have used to quantify these hydraulic capacities, namely leaf area-average xylem and phloem pressures, have some experimental relevance. For example, since most of the calculations were performed under the condition of constant transpiration, our results could be re-formulated as the experimentally acceptable definition of leaf conductance (Cochard et al., 2004; Zwieniecki et al., 2004; Sack and Holbrook, 2006; Prado and Maurel, 2013) by simply dividing the (constant) transpiration flux by the difference between the leaf-area average hydraulic pressure and the petiole pressure. Since the hydraulic pressure in the xylem has been determined for the coupled xylem-phloem system, the contributions of flows in both the xylem and the phloem have been included.

Based on our chosen indices, many interesting facts about the leaf response to factors such as leaf shape and vein angle come to light. These are discussed below. In this discussion it is necessary to keep in mind that the angiosperm model we have adopted, irrespective of the angle of the 2^nd^ order veins, remains that of a reticulate vein structure (vasculature) and is therefore exclusively pertinent to that of a dicot (eudicot, Rudall, 2007) leaf. Consequently, although we may in the discussion below speculate on implications for monocot leaves, the connection remains tenuous since leaves of true monocots have parallel vein structures connected to the petiole in a significantly different way. Here, we have not simulated monocot leaves.

We remark further that although convenient and of arguable experimental relevance, the boundary conditions of a stipulated constant transpiration rate in the xylem and a constant sucrose loading rate in the phloem network were somewhat artificial. Transpiration is firstly a time dependent phenomenon and secondly its magnitude is very much linked to the surrounding atmospheric condition. Setting a condition of given atmospheric pressure external to the leaf and monitoring the leaf's response in terms of the rate at which the leaf—as a whole—transpires would seem more in line with *in vivo* circumstances. But, as the focus of interest of the paper is on the internal movement of water we have chosen, for simplicity, to set the rate of transpiration as a boundary condition. Similarly, the production of photosynthetic compounds is also time dependent and a function of external variables (light intensity). However, here too we have simply set a fixed rate of production in order to explore the ramifications on the internal re-distribution of sucrose.

We should also point out that our study of the effect of 2^nd^ order vein angle was conducted under the constraint of constant total vein length. In this convolved system, we cannot alter vein angle at constant length without changing other structural variables (such as the spacing of connection points along the midrib vein and the total number of 2^nd^ order veins. On the other hand, by keeping the vein conductance and total vein length constant while altering the vein angle, we maintain the flow resistance through those veins; the hydrodynamic condition within the 2^nd^ veins remains unaffected (this is true as we have not included the feature of vein tapering).

To exhibit the contrast, in Supplementary Figure 15, we show the case where we have altered vein angle in the absence of the total length constraint, instead keeping fixed the total vein number and the spacing of branching or connecting points. The different conditions resulted, for the long leaf, in a monotonic increase in average xylem pressure. For shorter and wider leaves there is an optimal vein angle. Presumably, this arises from the fact that at large θ angles a significant area of the bottom part of the leaf is not serviced by 2^nd^ order veins, so water distribution to this region must be performed by the more highly resistive, higher order veins.

As a final general comment, in earlier papers by Foster and Miklavcic (Foster and Miklavcic, 2013, 2014) transport of water and solutes through the symplast and apoplast of living root tissues was treated as single pathway. Later efforts differentiated the two pathways Foster and Miklavcic (2013). In the present study of passive transport through parallel 2D networks, the leaf xylem forms part of the apoplast while the leaf phloem has been assumed to be solely the symplast. That is, we have modeled flow through the phloem as a single pathway when in real leaves the phloem (as well as surrounding living tissues such as the bundle sheath, sheath extension, the mesophyll, etc.) comprises both a symplastic pathway via cells that are interconnected by plasmadesmata and an apoplastic pathway through the porous cell wall region external to the contiguous plasmalemma network. The parameter values we have used to represent the conductivities of the phloem veins are therefore in some sense a weighted average of the conductances typical of the two phloem pathways. This idea has been discussed in detail in Foster and Miklavcic (2013). In a future model in which we include the mesophyll and other tissue elements we shall differentiate the two transport pathways.

For the same leaf shape (and vein conductivities) as that modeled by Cochard and co-workers (Cochard et al., 2004), we point out that our predicted hydraulic pressure distribution was qualitatively similar to their water potential distribution. Of course, since the detailed modeling of the vein network used in this study was somewhat different from that adopted in Cochard et al. (2004), the finer details of the distribution pattern were understandably slightly different.

We found the most important influencer of the xylem hydraulic pressure distribution to be the leaf shape. The leaf area-average xylem water pressure was lowest in the narrowest leaf, at all angles (Figure 7). Since the leaf areas and total vein lengths were fixed properties in all leaf shapes adopted in this study, this outcome cannot be attributed to differences in either total transpiration volume per unit time or total length of travel of water. This finding was true under all conditions studied, as evidenced also by Figures 10, 11 (e.g., see also Supplementary Figures 1, 4, 6, 9, 11).

With regard to the angle of 2^nd^ order veins, we have consistently found that at a vein angle within a small angular range roughly centered at 45° (Figure 7) the leaf area-average xylem hydraulic pressure is maximized. This finding was irrespective of external conditions of, say, transpiration and sucrose loading rates (Supplementary Figures 1, 4, 6, 8). However, we found that the response to vein angle exhibited a strong dependence on vein conductivity. For low 2^nd^ order vein conductances [lower than that adopted by Cochard et al. for a Laurel leaf (Cochard et al., 2004)], the response overall became less dependent on angle, indeed largely independent of angle in the range 0−45° from the perpendicular. Even beyond the upper limit of the latter range (at which the xylem pressure was a maximum) the dependence of the decay with angle was weaker (Figure 10). For high 2^nd^ order vein conductances, on the other hand, there was a strong linear dependence on angle up to a maximum pressure, which appeared at significantly larger angles [for the narrow leaf the angle at which the maximum occurred was 76° (Figure 11)]. Given other findings, these traits are relative the conductivities of higher order veins.

In the phloem, we have consistently found that the two-dimensional phloem pressure distribution showed an inverted behavior to that of the xylem hydraulic pressure distribution (Figure 4). A similar inverted response is also exhibited by the sucrose distribution (Figure 5). So, in a direct mirror reflection of the xylem hydraulic pressure, the narrower the leaf, the higher the phloem pressure, for all 2^nd^ order vein angles and under all the other conditions we've investigated, Figures 8, 9 (see also Supplementary Figures 2, 3, 7, 10, 12–14).

We infer from these results the important consequence that the angle of second-order veins, relative to the main vein, is a significant factor governing the efficient flow of water across a leaf, depending only on intrinsic vein properties such as the vein conductance and vein number and its connection with higher-order veins, but not on external factors such as transpiration rate or sucrose loading rate.

With our simulations were conveniently conducted at fixed rates of transpiration and sucrose loading (mostly uniformly across the leaf), it is possible to draw some further inferences.

To achieve the same constant rate of evaporation over the leaf, the xylem pressure must reach a much lower (more negative) value in the narrow leaf than in the case of the wider leaf case, This points to a less efficient xylem water transport system in narrow leaves. The (poor) efficiency is maximized when the veins are aligned roughly 45° to the main vein, although this depends on vein conductivity. The architecture is least efficient in transporting water when the 2^nd^ order veins are perpendicular to the main vein. Again, one imagines that narrow leaves would be more suited to having a parallel vein structure. Arguing similarly, the constant production of sucrose leads to a higher concentration distributed across the narrow leaf at steady state compared with the two wider leaves. This too suggests that narrow leaves are less effective in *passively* translocating photosynthates out through the petiole. Here, again it would be interesting to compare this narrow, reticulated leaf result with that of a monocot's parallel vein system to see whether there may be an evolutionary advantage of having the latter system in a leaf that is narrow.

As a final comment we might mention that one utility of a coupled xylem/phloem model is its potential to evaluate the degree to which water circulates between the xylem and the phloem networks. The degree to which plant nutrients are transported into the leaf in the xylem stream and delivered to sites in the phloem and mesophyll tissues will largely depend on this circulation flow. So, while membrane transporters of elemental nutrients are important to effect the transfer of nutrients into the symplast and hence living cells, the stream carrying the nutrients to membrane sites is critically important (Yamaji and Ma, 2014). Although we have not fully investigated this aspect here, our model represents an essential step to understanding this complex circulatory dynamic. In Supplementary Figure 16, we show an area map of the difference between xylem water hydraulic pressure and phloem total pressure. The difference is non-uniform with the xylem pressure generally higher than the total phloem pressure particularly near the leaf petiole. The leaf area-average pressure difference is shown in Supplementary Figure 17). Any further comments will have to wait until for a more representative model of the complicated dynamics of water and solutes in the xylem and phloem (Foster and Miklavcic, 2016, 2017; Sakurai et al., 2017).

## 5. Concluding Remarks

Despite the model resources developed in this work, it is not possible to draw authoritative conclusions on the evolutionary implications of leaf shape. As such, we cannot speculate on some questions such as why a leaf is broad or narrow. It is well-recognized that a very broad spectrum of leaf shapes in the angiosperm class of plants exist. The shape of leaves is a result of a complex combination, cooperation and competition between various external and internal influences. Which factor is most influential, if any, in determining the evolution of a given shape cannot be addressed here. For the moment, our model does not incorporate important external factors such as degree and duration of light intensity, temperature, air moisture, etc. On the other hand, what we can do is speculate, for a given shape, on what vein arrangement is optimal. In this regard our investigation has complemented the many early studies leaf shape evolution that have focused more on the edge shape of leaves, but also on the vasculature system (Cochard et al., 2004; Sack and Frole, 2006; McKown et al., 2010; Nicotra et al., 2011; Sommerville et al., 2012; Tsukaya, 2018). Those studies did not consider the contribution of vein angle, nor did they indicate that it could be one of the more important geometric characteristic. We believe that our model study contributes to the body of knowledge and improved understanding of how vein angles feature in an evolutionary perspective.

On the whole, our results suggest that it may be of some evolutionary hydraulic advantage for plants to have broader leaves. As for narrow leaves it appears that the disadvantage can be minimized if the 2^nd^ order veins (at least) are both highly conductive and arranged at small acute angles to the main vein. If the alignment is too great, however, (approaching a parallel vein system) the efficiency again decreases. Although we did not model the parallel vein system itself, one can conjecture that from this point a narrow leaf might well be better suited to have more a parallel 2^nd^ order vein arrangement.

In a future exercise it should be possible to compare our theoretical results with those measured on real leaves, although a comparison would require a good deal of quantitative anatomical and physiological information such as vein network structure and hierarchy and vein conductances. One means of bringing a comparison about would involve a series of measurements of water potential as a function of transpiration (and other external conditions) across many different species, and for us to compare our representative calculations based on the quantified vein architectures of those species. Alternatively, based on the higher level predictions of our model, we imagine the possibility of a collecting information on leaf size and shape, and leaf vein architecture from leaves from different locations around the world, and then correlate this information with the climate at their origin to see if our general conclusions (such as long thin veins having more acute 2^nd^ order vein angles) are verified.

The model we have utilized in this work presents a relatively simple description of actual vein architecture. No account has been taken of details such as the curving and tapering of veins, which are features of eucamptodromous venation. Moreover, branched veins as in craspedodromous and actinodromous vascular systems (Hickey, 1973) were not considered in this study. Secondly, we only simulated systems using (mostly) the vein conductivities referred to in the works of Cochard et al. (2004), although other 2^nd^ order vein conductivities were also considered. But to properly understand the functionality and evolution of angiosperm vein systems, we aim in our future investigations to entertain more accurate vascular architectures with measured vein conductivities for different species. Nevertheless, even with the simple geometric vein arrangement we have employed in the model that we have presented we are able to show some interesting consequences of leaf venation. The present study is our initial attempt toward understanding the evolution of the complicated vein systems and leaf shapes of plants. In future work, the model will be improved to also feature more tissue-specific details such as the bundle sheath surrounding veins and the mesophyll. That level of elaboration is needed in order to include a more detailed representation of both symplastic and apoplastic pathways for water and solutes.

## Data Availability Statement

The data underpinning the results presented in figures herein are available from the authors upon request.

## Author Contributions

The authors were equal contributors to the design of simulation experiments, analysis of results and drafting of the paper. SM designed the model. GS performed the simulations.

## Conflict of Interest

The authors declare that the research was conducted in the absence of any commercial or financial relationships that could be construed as a potential conflict of interest.

## References

[B1] Batchelor G. K. (1967). An Introduction to Fluid Dynamics. Cambridge: Cambridge University Press.

[B2] Boersma L. Lindstrom F. Childs S. (1991). Model for steady state coupled transport in xylem and phloem. Agronomy J. 83, 401–408. 10.2134/agronj1991.00021962008300020028x

[B3] Brodersen C. R. Roark L. C. Pittermann J. (2012). The physiological implications of primary xylem organization in two ferns. Plant Cell Environ. 35, 1898–1911. 10.1111/j.1365-3040.2012.02524.x 22524854

[B4] Cochard H. Nardini A. Coll L. (2004). Hydraulic architecture of leaf blades: where is the main resistance? Plant Cell Environ. 27, 1257–1267. 10.1111/j.1365-3040.2004.01233.x

[B5] Daudet F. A. Lacointe A. Gaudillere J. Cruiziat P. (2002). Generalized Münch coupling between sugar and water fluxes for modelling carbon allocation as affected by water status. J. Theoret. Biol. 214, 481–498. 10.1006/jtbi.2001.2473 11846604

[B6] Esau K. (1953). Plant Anatomy. New York, NY: John Wiley and Sons 10.1097/00010694-195305000-00014

[B7] Foster K. J. Miklavcic S. J. (2013). Mathematical modelling of the uptake and transport of salt in plant roots. J. Theoret. Biol. 336, 132–143. 10.1016/j.jtbi.2013.07.025 23916880

[B8] Foster K. J. Miklavcic S. J. (2014). On the competitive uptake and transport of ions through differentiated root tissues. J. Theoret. Biol. 340, 1–10. 10.1016/j.jtbi.2013.09.004 24036203

[B9] Foster K. J. Miklavcic S. J. (2016). Modeling root zone effects on preferred pathways for the passive transport of ions and water in plant roots. Front. Plant Sci. 7:914. 10.3389/fpls.2016.00914 27446144 PMC4917552

[B10] Foster K. J. Miklavcic S. J. (2017). A comprehensive biophysical model of ion and water transport in plant roots. I. Clarifying the roles of endodermal barriers in the salt stress response. Front. Plant Sci. 8:1326. 10.3389/fpls.2017.01326 28804493 PMC5532442

[B11] Foster K. J. Miklavcic S. J. (2019). A comprehensive biophysical model of ion and water transport in plant roots. II. Clarifying the roles of sos1 in the salt stress response in arabidopsis. Front. Plant Sci. 10:1121. 10.3389/fpls.2019.01121 31620152 PMC6759596

[B12] Goeschl J. D. Magnuson C. Demichele D. W. Sharpe P. J. (1976). Concentration-dependent unloading as a necessary assumption for a closed form mathematical model of osmotically driven pressure flow in phloem. Plant Physiol. 58, 556–562. 10.1104/pp.58.4.556 16659717 PMC543281

[B13] Hickey L. J. (1973). Classification of the architecture of dicotyledonous leaves. Am. J. Bot. 60, 17–33. 10.1002/j.1537-2197.1973.tb10192.x

[B14] Hölttä T. Mencuccini M. Nikinmaa E. (2009). Linking phloem function to structure: analysis with a coupled xylem-phloem transport model. J. Theoret. Biol. 259, 325–337. 10.1016/j.jtbi.2009.03.039 19361530

[B15] Hölttä T. Vesala T. Sevanto S. Perämäki M. Nikinmaa E. (2006). Modeling xylem and phloem water flows in trees according to cohesion theory and Münch hypothesis. Trees 20, 67–78. 10.1007/s00468-005-0014-6

[B16] Katchalsky A. Curran P. F. (1965). Nonequilibrium Thermodynamics in Biophysics. Cambridge, MA: Harvard University Press.

[B17] Kramer P. J. Boyer J. S. (1995). Water Relations of Plants and Soils. San Diego, CA: Academic Press 10.1016/B978-012425060-4/50003-6

[B18] Lacointe A. Minchin P. E. (2008). Modelling phloem and xylem transport within a complex architecture. Funct. Plant Biol. 35, 772–780. 10.1071/FP08085 32688831

[B19] Lhomme J.-P. Rocheteau A. Ourcival J. Rambal S. (2001). Non-steady-state modelling of water transfer in a mediterranean evergreen canopy. Agric. Forest Meteorol. 108, 67–83. 10.1016/S0168-1923(01)00218-0

[B20] Martre P. Cochard H. Durand J.-L. (2001). Hydraulic architecture and water flow in growing grass tillers (*Festuca arundinacea* Schreb.). Plant Cell Environ. 24, 65–76. 10.1046/j.1365-3040.2001.00657.x

[B21] Mathworks (2020). MATLAB version 9.8.0.1323502 (R2020a). Natick, MA: The Mathworks, Inc.

[B22] McKown A. D. Cochard H. Sack L. (2010). Decoding leaf hydraulics with a spatially explicit model: principles of venation architecture and implications for its evolution. Am. Natural. 175, 447–460. 10.1086/650721 20178410

[B23] Meinzer F. Goldstein G. Neufeld H. Grantz D. Crisosto G. (1992). Hydraulic architecture of sugarcane in relation to patterns of water use during plant development. Plant Cell Environ. 15, 471–477. 10.1111/j.1365-3040.1992.tb00998.x

[B24] Meinzer F. Grantz D. (1990). Stomatal and hydraulic conductance in growing sugarcane: stomatal adjustment to water transport capacity. Plant Cell Environ. 13, 383–388. 10.1111/j.1365-3040.1990.tb02142.x

[B25] Nicotra A. B. Leigh A. Boyce C. K. Jones C. S. Niklas K. J. Royer D. L.. (2011). The evolution and functional significance of leaf shape in the angiosperms. Funct. Plant Biol. 38, 535–552. 10.1071/FP11057 32480907

[B26] Nikinmaa E. Hölttä T. Hari P. Kolari P. Mäkelä A. Sevanto S.. (2013). Assimilate transport in phloem sets conditions for leaf gas exchange. Plant Cell Environ. 36, 655–669. 10.1111/pce.12004 22934921

[B27] North G. B. Lynch F. H. Maharaj F. D. Phillips C. A. Woodside W. T. (2013). Leaf hydraulic conductance for a tank bromeliad: axial and radial pathways for moving and conserving water. Front. Plant Sci. 4:78. 10.3389/fpls.2013.00078 23596446 PMC3622035

[B28] Prado K. Maurel C. (2013). Regulation of leaf hydraulics: from molecular to whole plant levels. Front. Plant Sci. 4:255. 10.3389/fpls.2013.00255 23874349 PMC3711007

[B29] Roth-Nebelsick A. Uhl D. Mosbrugger V. Kerp H. (2001). Evolution and function of leaf venation architecture: a review. Ann. Bot. 87, 553–566. 10.1006/anbo.2001.1391

[B30] Rudall P. (2007). Anatomy of Flowering Plants. Cambridge: Cambridge University Press 10.1017/CBO9780511801709

[B31] Sack L. Frole K. (2006). Leaf structural diversity is related to hydraulic capacity in tropical rain forest trees. Ecology 87, 483–491. 10.1890/05-0710 16637372

[B32] Sack L. Holbrook N. M. (2006). Leaf hydraulics. Annu. Rev. Plant Biol. 57, 361–381. 10.1146/annurev.arplant.56.032604.144141 16669766

[B33] Sack L. Scoffoni C. (2013). Leaf venation: structure, function, development, evolution, ecology and applications in the past, present and future. N. Phytol. 198, 983–1000. 10.1111/nph.12253 23600478

[B34] Sakurai G. Yamaji N. Mitani-Ueno N. Yokozawa M. Ono K. Ma J. F. (2017). A model of silicon dynamics in rice: an analysis of the investment efficiency of si transporters. Front. Plant Sci. 8:1187. 10.3389/fpls.2017.01187 28744291 PMC5504195

[B35] Seki M. Feugier F. G. Song X.-J. Ashikari M. Nakamura H. Ishiyama K.. (2015). A mathematical model of phloem sucrose transport as a new tool for designing rice panicle structure for high grain yield. Plant Cell Physiol. 56, 605–619. 10.1093/pcp/pcu191 25516572

[B36] Sommerville K. E. Sack L. Ball M. C. (2012). Hydraulic conductance of acacia phyllodes (foliage) is driven by primary nerve (vein) conductance and density. Plant Cell Environ. 35, 158–168. 10.1111/j.1365-3040.2011.02425.x 21923760

[B37] Steppe K. De Pauw D. J. Lemeur R. Vanrolleghem P. A. (2006). A mathematical model linking tree sap flow dynamics to daily stem diameter fluctuations and radial stem growth. Tree Physiol. 26, 257–273. 10.1093/treephys/26.3.257 16356899

[B38] Thompson M. V. Holbrook N. M. (2003). Application of a single-solute non-steady-state phloem model to the study of long-distance assimilate transport. J. Theoret. Biol. 220, 419–455. 10.1006/jtbi.2003.3115 12623280

[B39] Tsukaya H. (2018). “A consideration of leaf shape evolution in the context of the primary function of the leaf as a photosynthetic organ,” in The Leaf: A Platform for Performing Photosynthesis, eds W. W. Adams III and I. Terashima (Berlin: Springer), 1–26. 10.1007/978-3-319-93594-2_1

[B40] Yamaji N. Ma J. F. (2014). The node, a hub for mineral nutrient distribution in graminaceous plants. Trends Plant Sci. 19, 556–563. 10.1016/j.tplants.2014.05.007 24953837

[B41] Zwieniecki M. A. Boyce C. K. Holbrook N. M. (2004). Functional design space of single-veined leaves: role of tissue hydraulic properties in constraining leaf size and shape. Ann. Bot. 94, 507–513. 10.1093/aob/mch173 15319225 PMC4242227

[B42] Zwieniecki M. A. Melcher P. J. Boyce C. K. Sack L. Holbrook N. M. (2002). Hydraulic architecture of leaf venation in *Laurus nobilis* L. Plant Cell Environ. 25, 1445–1450. 10.1046/j.1365-3040.2002.00922.x

[B43] Zwieniecki M. A. Stone H. A. Leigh A. Boyce C. K. Holbrook N. M. (2006). Hydraulic design of pine needles: one-dimensional optimization for single-vein leaves. Plant Cell Environ. 29, 803–809. 10.1111/j.1365-3040.2005.01448.x 17087464

