# Supplementary material for: On the Efficacy of Water Transport in Leaves. A Coupled Xylem-Phloem Model of Water and Solute Transport
Source: Front Plant Sci. 2021 Feb 4;12:615457. doi: 10.3389/fpls.2021.615457 (PMC7889512; doi:10.3389/fpls.2021.615457)
Supplement: Supplementary file 1 [file Data_Sheet_1.PDF]

Supplementary Material: On the efficacy of water transport in leaves. A coupled xylem-phloem model of water and solute transport

1 SUPPLEMENTARY FIGURES

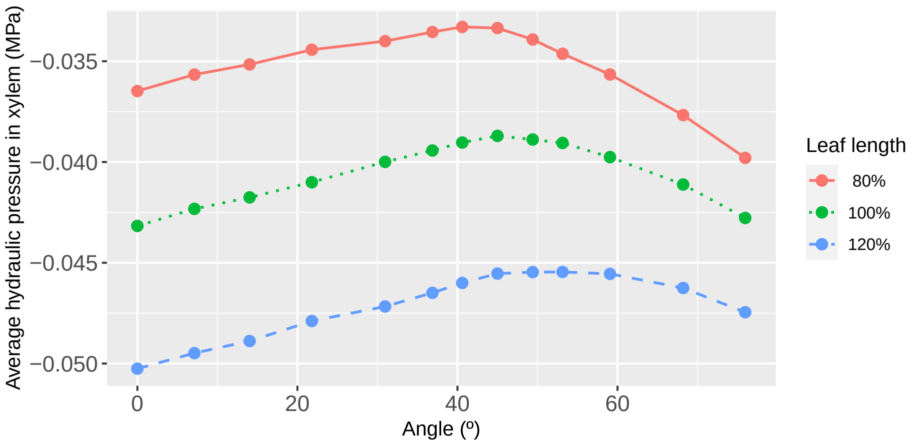

Figure S1. The functional relationship between leaf area-average, xylem hydraulic pressure and second-order vein angle. Curve descriptions and simulation conditions are as in Figure 7 in the main text except with a evaporation rate of -1.00 mmol s^{-1} m^{-2} (*i.e.* a factor of one half of the core value).

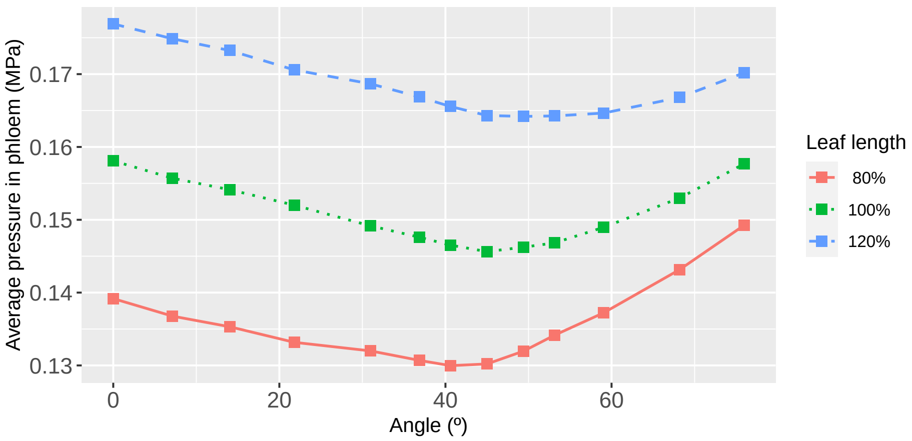

Figure S2. The functional relationship between leaf area-average, phloem hydraulic pressure and second-order vein angle. Curve descriptions and simulation conditions are as in Figure 7 in the main text except with a evaporation rate of -1.00 mmol s^{-1} m^{-2} (*i.e.* a factor of one-second that of the core value).

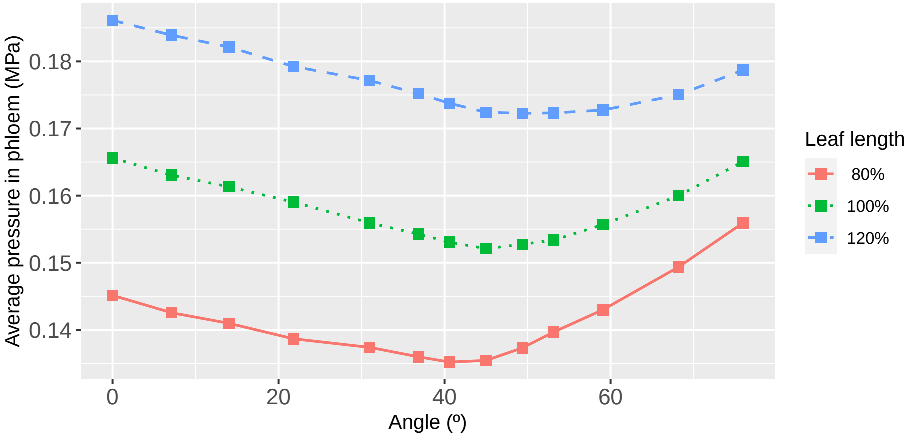

Figure S3. The functional relationship between leaf area-average, phloem hydraulic pressure and second-order vein angle. Curve descriptions and simulation conditions are as in Figure 7 in the main text except with an evaporation rate of 0.00 mmol s^{-1} m^{-2} (*i.e.* no evaporation).

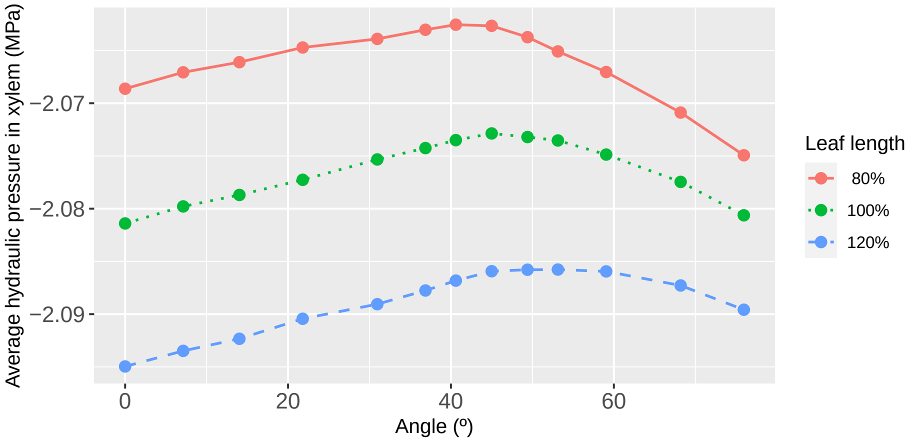

Figure S4. The functional relationship between leaf area-average, xylem hydraulic pressure and second-order vein angle. Curve descriptions and simulation conditions are as in Figure 7 in the main text except with a hydraulic pressure in the xylem of -2.00 MPa and a hydraulic pressure in the phloem of -1.80 MPa.

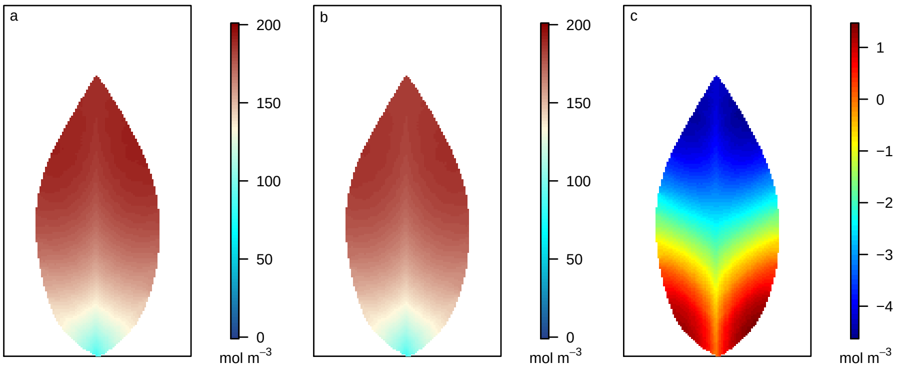

Figure S5. Leaf area distribution of sucrose concentration in the phloem network. Panel (a) depicts the leaf with base setting, (b) shows the leaf under a negative linear gradient in the sucrose loading distribution of -50.00% (with the sucrose loading rate higher at the petiole), while (c) shows the difference in the sucrose concentration between these two leaves. In addition, the second-order veins are aligned at 45° to the main vein (*i.e.*, 45° to the reference state of perpendicular veins).

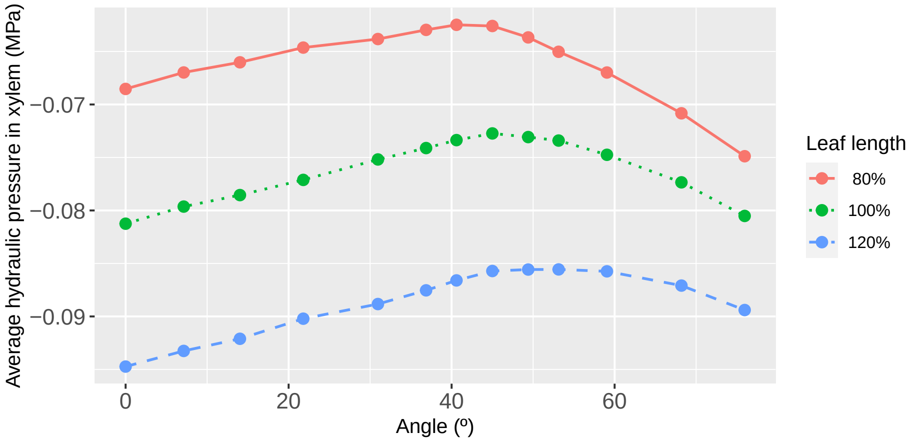

Figure S6. The functional relationship between leaf area-average, xylem hydraulic pressure and second-order vein angle. Curve descriptions and simulation conditions are as in Figure 7 in the main text except with a negative linear gradient in the sucrose loading distribution of -50.00% (with the sucrose loading rate higher at the petiole).

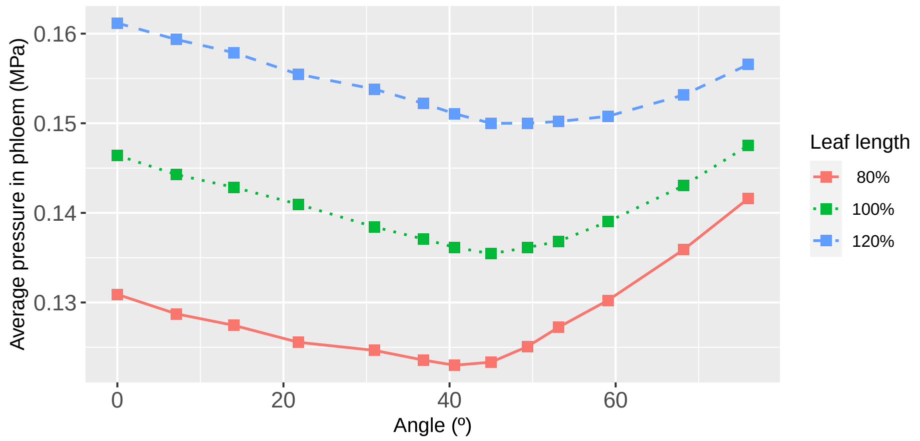

Figure S7. The functional relationship between leaf area-average, phloem hydraulic pressure and second-order vein angle. Curve descriptions and simulation conditions are as in Figure 7 in main text except with a negative linear gradient of the sucrose distribution of -50.00% (with the sucrose loading rate higher at the petiole).

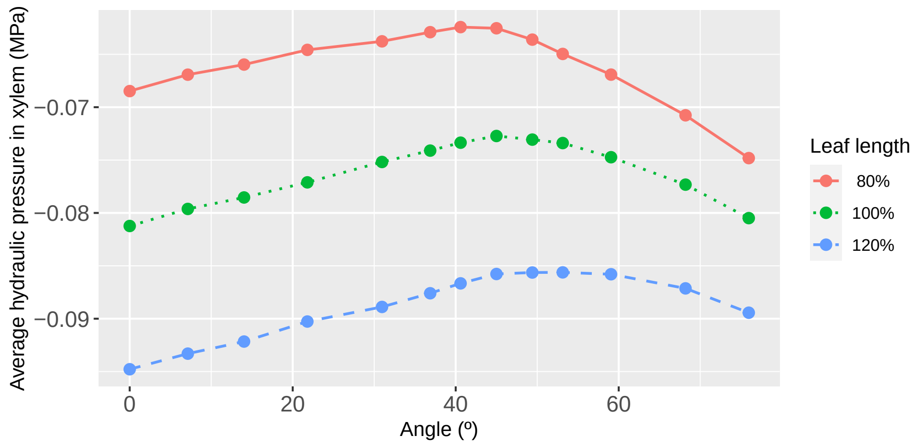

Figure S8. The functional relationship between leaf area-average, xylem hydraulic pressure and second-order vein angle. Curve descriptions and simulation conditions are as in Figure 7 in the main text except with a phloem/xylem conductance (K^{ph}_{ij-c}) of 0.0005 mmol s^{-1} MPa^{-1} (*i.e.* a factor of one-thousandth that of the core value).

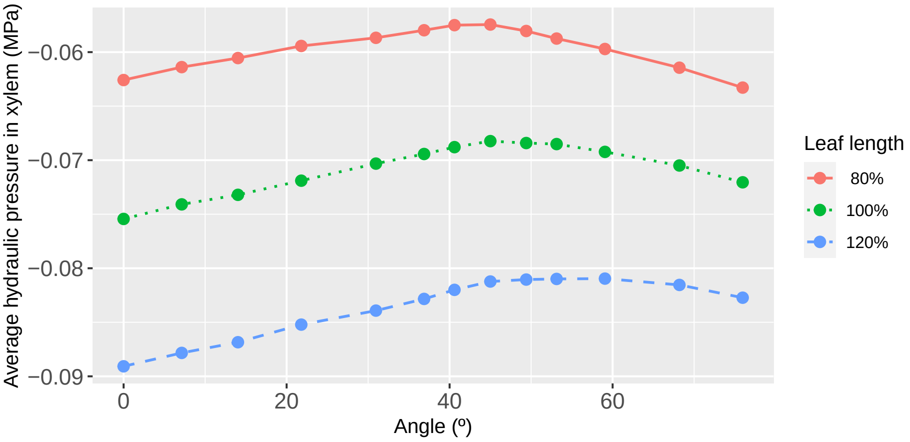

Figure S9. The functional relationship between leaf area-average, xylem hydraulic pressure and second-order vein angle. Curve descriptions and simulation conditions are as in Figure 7 in the main text except with a higher conductance of 5th order veins (*i.e.* 5th order veins are replaced by 4th order veins).

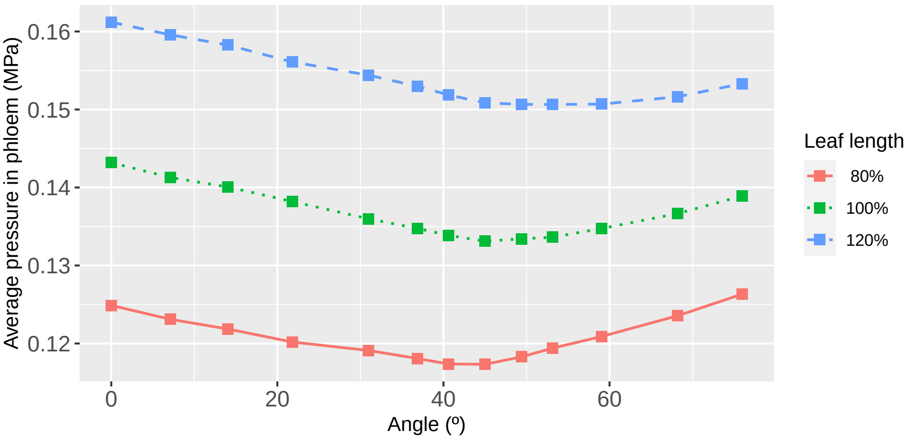

Figure S10. The functional relationship between leaf area-average, phloem hydraulic pressure and second-order vein angle. Curve descriptions and simulation conditions are as in Figure 7 in main text except with a higher conductance of 5th order veins (*i.e.* 5th order veins replaced by 4th order veins).

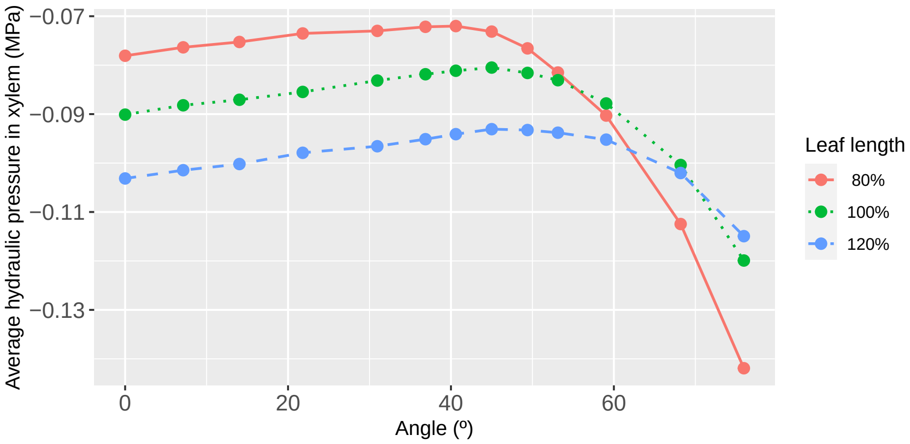

Figure S11. The functional relationship between leaf area-average, xylem hydraulic pressure and second-order vein angle. Curve descriptions and simulation conditions are as in Figure 7 in main text except with a lower conductance of 4th order veins (*i.e.* 4th order veins replaced by 5th order veins).

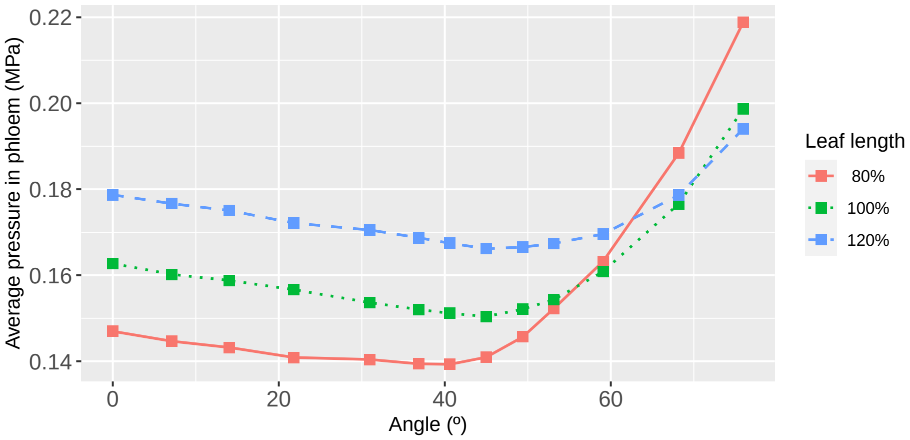

Figure S12. The functional relationship between leaf area-average, phloem hydraulic pressure and second-order vein angle. Curve descriptions and simulation conditions are as in Figure 7 in the main text except with a lower conductance of 4th order veins (*i.e.* 4th order veins replaced by 5th order veins).

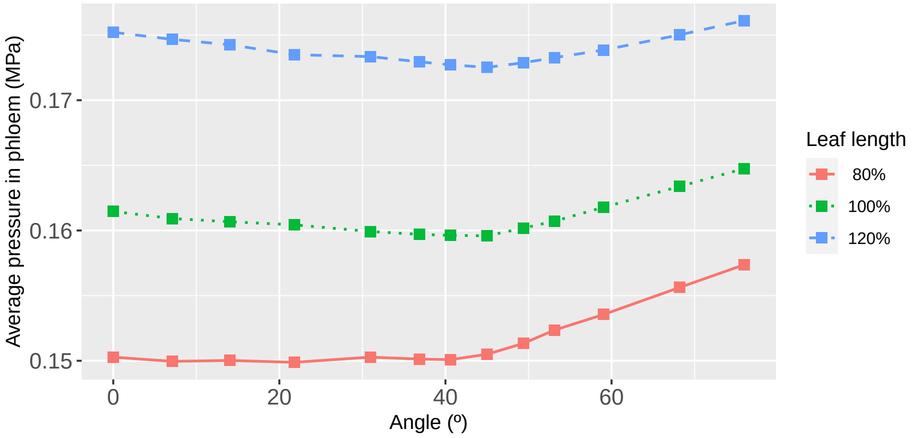

Figure S13. The functional relationship between leaf area-average, phloem hydraulic pressure and second-order vein angle. Curve descriptions and simulation conditions are as in Figure 7 in the main text except with a uniform second-order vein conductance of $K^{xyl} = 100K^{ph}/3 = 1 \times 10^{-4}$ mmol s^{-1} m^{-2} (*i.e.* a factor of one-fifth that of the core value).

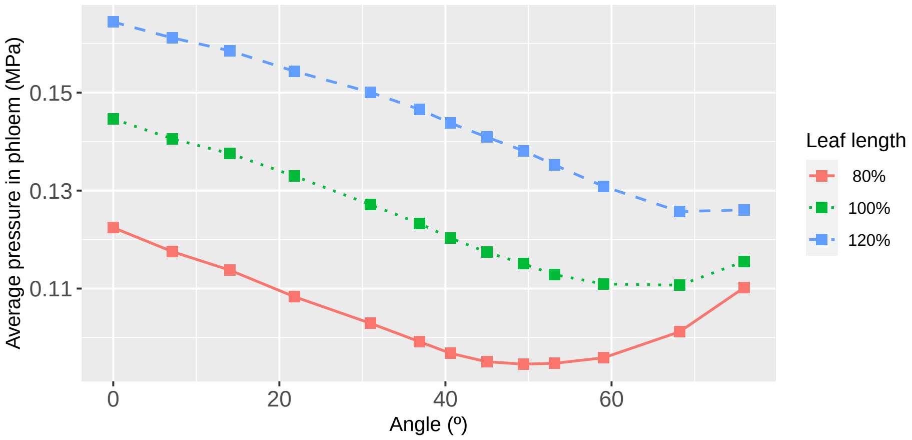

Figure S14. The functional relationship between leaf area-average, phloem hydraulic pressure and second-order vein angle. Curve descriptions and simulation conditions are as in Figure 7 in main text except with a uniform second-order vein conductance of $K^{xyl} = 100K^{ph}/3 = 2.5 \times 10^{-3}$ mmol s^{-1} m^{-2} (*i.e.* an increase by a factor of 5 of the core value).

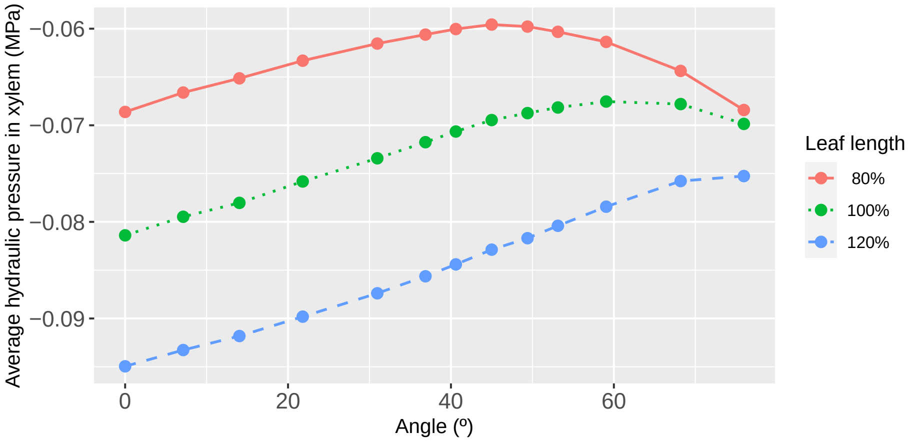

Figure S15. The functional relationship between leaf area-average, xylem hydraulic pressure and second-order vein angle. Curve descriptions and simulation conditions are as in Figure 7 in the main text except with a fixed total vein number and fixed spacing of branching points (*i.e.*, in the absence of the constraint of fixed total 2nd order vein length).

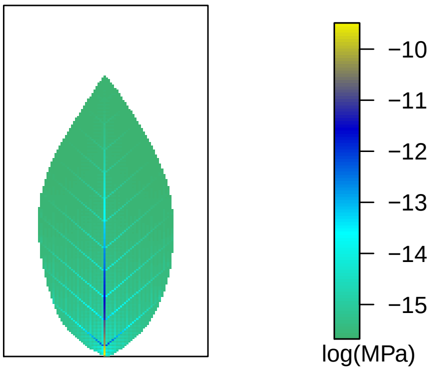

Figure S16. Leaf area distribution of the difference between the xylem hydraulic pressure and the phloem total pressure in the leaf with 2nd order veins aligned 45° to the main vein. Notice that the legend is log(MPa) to improve readability.

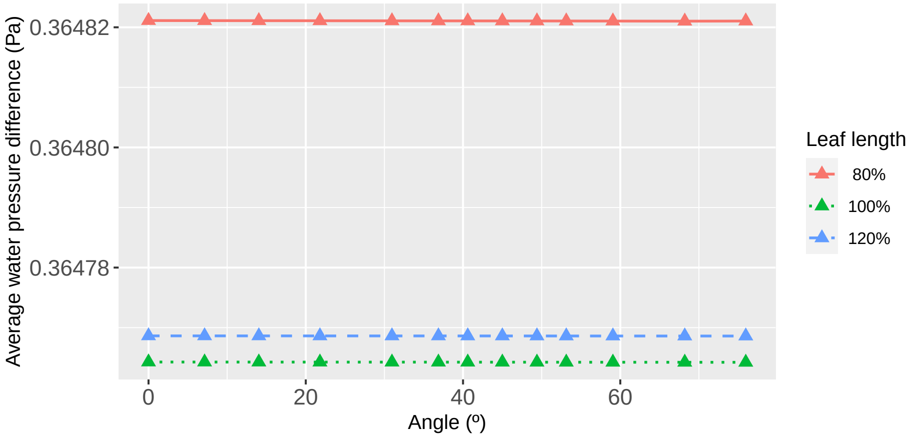

Figure S17. The functional relationship between leaf area-average, the pressure difference (between the xylem hydraulic pressure and the phloem total pressure) and second-order vein angle. Curve descriptions and simulation conditions are as in Figure 7 in the main text.
